# Comparative genomics of *Thermus thermophilus *and *Deinococcus radiodurans*: divergent routes of adaptation to thermophily and radiation resistance

**DOI:** 10.1186/1471-2148-5-57

**Published:** 2005-10-20

**Authors:** Marina V Omelchenko, Yuri I Wolf, Elena K Gaidamakova, Vera Y Matrosova, Alexander Vasilenko, Min Zhai, Michael J Daly, Eugene V Koonin, Kira S Makarova

**Affiliations:** 1 Department of Pathology, F.E. Hebert School of Medicine, Uniformed Services University of the Health Sciences, Bethesda, MD 20814-4799, USA; 2 National Center for Biotechnology Information, National Library of Medicine, National Institutes of Health, Bethesda, MD 20894, USA

## Abstract

**Background:**

*Thermus thermophilus *and *Deinococcus radiodurans *belong to a distinct bacterial clade but have remarkably different phenotypes. *T. thermophilus *is a thermophile, which is relatively sensitive to ionizing radiation and desiccation, whereas *D. radiodurans *is a mesophile, which is highly radiation- and desiccation-resistant. Here we present an in-depth comparison of the genomes of these two related but differently adapted bacteria.

**Results:**

By reconstructing the evolution of *Thermus *and *Deinococcus *after the divergence from their common ancestor, we demonstrate a high level of post-divergence gene flux in both lineages. Various aspects of the adaptation to high temperature in *Thermus *can be attributed to horizontal gene transfer from archaea and thermophilic bacteria; many of the horizontally transferred genes are located on the single megaplasmid of *Thermus*. In addition, the *Thermus *lineage has lost a set of genes that are still present in *Deinococcus *and many other mesophilic bacteria but are not common among thermophiles. By contrast, *Deinococcus *seems to have acquired numerous genes related to stress response systems from various bacteria. A comparison of the distribution of orthologous genes among the four partitions of the *Deinococcus *genome and the two partitions of the *Thermus *genome reveals homology between the *Thermus *megaplasmid (pTT27) and *Deinococcus *megaplasmid (DR177).

**Conclusion:**

After the radiation from their common ancestor, the *Thermus *and *Deinococcus *lineages have taken divergent paths toward their distinct lifestyles. In addition to extensive gene loss, *Thermus *seems to have acquired numerous genes from thermophiles, which likely was the decisive contribution to its thermophilic adaptation. By contrast, *Deinococcus *lost few genes but seems to have acquired many bacterial genes that apparently enhanced its ability to survive different kinds of environmental stresses. Notwithstanding the accumulation of horizontally transferred genes, we also show that the single megaplasmid of *Thermus *and the DR177 megaplasmid of *Deinococcus *are homologous and probably were inherited from the common ancestor of these bacteria.

## Background

*Deinococcus spp*. and *Thermus spp*. are believed to belong to a distinct branch of bacteria called the *Deinococcus-Thermus *group [1]. The common origin of these bacteria is supported by the fact that they consistently form a strongly supported clade in phylogenetic trees of ribosomal RNAs and several conserved proteins including ribosomal proteins, RNA polymerase subunits, RecA, and others [1-7]. The *Thermus *genus currently consists of 14 thermophilic species, whereas the *Deinococcus *genus includes at least eleven, mostly mesophilic species known for their extreme resistance to γ-irradiation and other agents causing DNA damage, particularly, desiccation [7,8]. Interestingly, two new species, *D. frigens sp. nov., D. saxicola*, have been isolated recently from Antarctic rock and soil samples [9]. *D. geothermalis *and *D. murrayi*, are considered to be thermophilic (***t***_opt_~45–50°C) [10].

*T. thermophilus *(TT) (strain HB27, ATCC BAA-163)and *D. radiodurans *(DR) (strain R1, ATCC BAA-816) have similar general physiology, both being catalase-positive, red-pigmented, non-sporulating, aerobic chemoorganoheterotrophs [11]. However, the two organisms are dramatically different in terms of stress resistance: DR is one of the most resistant to radiation and desiccation among the characterized organisms and, generally, can survive diverse types of oxidative stress, whereas TT is a thermophile that thrives under thermal stress conditions but is relatively sensitive to radiation and other forms of oxidative stress. Recently, the genomes of *T. thermophilus *HB27 and HB8 [12,13] became available for comparison with the previously sequenced genome of *D. raduodurans *R1 [14].

Despite extensive research, genetic systems underlying both thermophilic adaptations and radiation resistance remain poorly understood. Attempts have been made to detect "thermophilic determinants" in the proteomes of thermophiles using a comparative-genomic approach. This resulted in the delineation of a set of proteins that might be associated with the thermophilic phenotype, although most of these are significantly enriched in thermophiles but not unique to these organisms [15-17]. Other distinctions between thermophilic and mesophilic proteins might be due to differences in their structural properties, such as different amino acid compositions, loop lengths, number of salt bridges, strength of hydrophobic interaction, number of disulfide bonds, and other features [18-25].

Various hypotheses also have been proposed to explain radiation resistance, some postulating the existence of specialized genetic systems, particularly those for DNA repair and stress response [7,26]. Recently, however, alternative possibilities have been advanced. For example, the post-irradiation adjustment of metabolism of *D. radiodurans *might prevent production of reactive oxygen species (ROS) by decreasing the number of reactions involving oxygen [27,28], and high intracellular manganese concentrations of *Deinococcus *spp. might help scavenge ROS generated during irradiation and post-irradiation recovery [28,29]. However, these explanations of radiation resistance have received little direct support from comparative-genomic analyses [7,27,28].

Several evolutionary processes could potentially contribute to the genome differentiation of TT and DR subsequent to the divergence from the common ancestor: (i) differential gene loss and gain, (ii) acquisition of genes via horizontal gene transfer (HGT) which may be followed by loss of the ancestral orthologous gene (xenologous gene displacement (XGD)), (iii) lineage-specific expansion of paralogous gene families by duplication and/or acquisition of paralogs via HGT; (IV) modification of amino acid composition that could affect protein stability. Here, we experimentally characterize radiation and desiccation resistance of TT in comparison to DR, and, assess the contribution of different evolutionary processes to distinct adaptations of TT and DR, using a variety of comparative-genomic approaches and phylogenetic analysis. We identify the unique feature of the gene repertoires of TT and DR that might contribute to these phenotypic differences, which could be the subject of further experimental work. In addition, we describe the results of a detailed analysis of the proteins predicted to be involved in DNA repair and stress response functions, which are particularly relevant for adaptive evolution of resistance phenotypes.

## Results and discussion

### Experimental characterization of resistance to gamma-radiation and desiccation, and determination of intracellular Mn/Fe ratio for *T. thermophilus*

**Figure 1:**
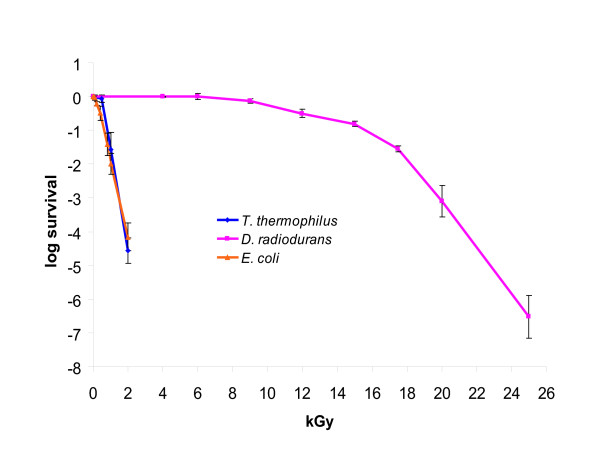
Radiation resistance of *T. thermophilus *(ATCC BAA-163), *D. radiodurans *(ATCC BAA-816) and *E. coli *(K-12 MG1655, provided by M. Cashel, NIH) (^60^Co irradiation). Standard deviations for the data points are shown.

**Figure 2:**
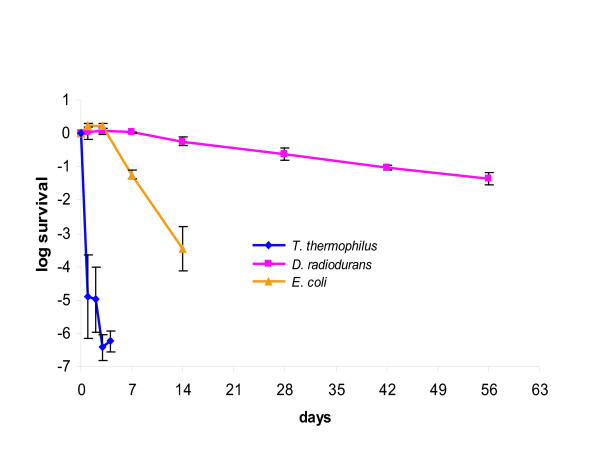
Desiccation resistance of *T. thermophilus *(ATCC BAA-163), *D. radiodurans *(ATCC BAA-816) and *E. coli *(K-12 MG1655) (room temperature). Note that no surviving cells were obtained for cells desiccated at 65°C. Standard deviations for five data points are shown.

While the radiation- and desiccation-resistant phenotypes of DR and the thermal requirements of both TT and DR have been studied extensively ([7,12,30] and references therein), we are unaware of any detailed characterization of the response of TT to irradiation or desiccation. Therefore, we sought to investigate these properties in order to obtain a more complete picture of the differences in the stress response phenotypes of TT and DR. Not unexpectedly, we found that TT was much more sensitive to acute irradiation than DR. The survival curve of TT is similar to that of *Esherichia coli *K12. For DR, the radiation dose yielding 10% colony forming unit (CFU) survival (D_10_) is ~16 kGy, whereas for TT and *E. coli*, the D_10 _dose is ~0.8 kGy and ~0.7 kGy, respectively (Figure 1). TT is also highly sensitive to desiccation. The 10% CFU desiccation survival frequency of DR is sustained after 30 days, while TT reaches the 10% CFU desiccation survival at ~10 hours (0.4% survival after 24 hours) and, by the 5th day, suffers essentially 100% lethality. The low resistance of TT to desiccation is observed regardless of the temperature and drying rate (see Additional file 1, "Desiccation of TT at 65°C"). The desiccation resistance of *E. coli *was found to be intermediate between those of TT and DR (Figure 2).

We recently reported a trend of intracellular Mn/Fe concentration ratios in bacterial IR resistance, where very high and very low Mn/Fe ratios correlated with very high and very low resistances, respectively [29]. We have also shown that growing *D. radiodurans *in conditions which limited Mn(II) accumulation, significantly lowered the cells' IR resistance [29]. These observations led to the hypothesis that the ratio of Mn to Fe in a cell might determine the relative abundance of different ROS induced during exposure to and recovery from IR [28,29]. At high concentrations, Mn(II) can act as true catalyst of the dismutation of superoxide (O_2_^•-^), with Mn cycling between the divalent and trivalent states; Mn redox-cycling scavenges both O_2_^•- ^and hydrogen peroxide [31]. In this context, we determined the intracellular Mn/Fe ratio of TT using an inductively coupled plasma-mass spectrometry method (ICP-MS), as previously described [29]. TT cells contained 0.211 (± 0.0254) nmol Mn/mg protein and 4.54 (± 0.778) nmol Fe/mg protein. The intracellular Mn/Fe ratio of TT (D_10_, ~0.8 kGy) is 0.047 compared to 0.0072 for *E. coli *(D_10_, ~0.7 kGy), 0.24 for *D. radiodurans *(D_10_, ~16 kGy), and <0.0001 for *Pseudomonas putida *(D_10_, ~0.25 kGy) [29]. Thus, TT appears to be somewhat more sensitive to acute IR and desiccation than predicted by its Mn/Fe ratio, suggesting the possibility of more complex relationships between these two variables. Notably, scavenging of O_2_^•- ^by Mn(II) is highly dependent on the availability of H_2_O_2_, which in TT would be expected to become limiting during recovery at 65°C because of thermal decomposition [32]; inefficient Mn redox-cycling can lead to Mn(III) accumulation, which is cytotoxic. Both DR and TT encode an ABC- type Mn transporter and a transcriptional regulator that probably regulates Mn homeostasis. Additionally, DR has a NRAMP family Mn transporter, for which there is no ortholog in TT.

### Reconstruction of the gene-content tree and the gene repertoire of the common ancestor of *Deinococcus and Thermus*

**Figure 3:**
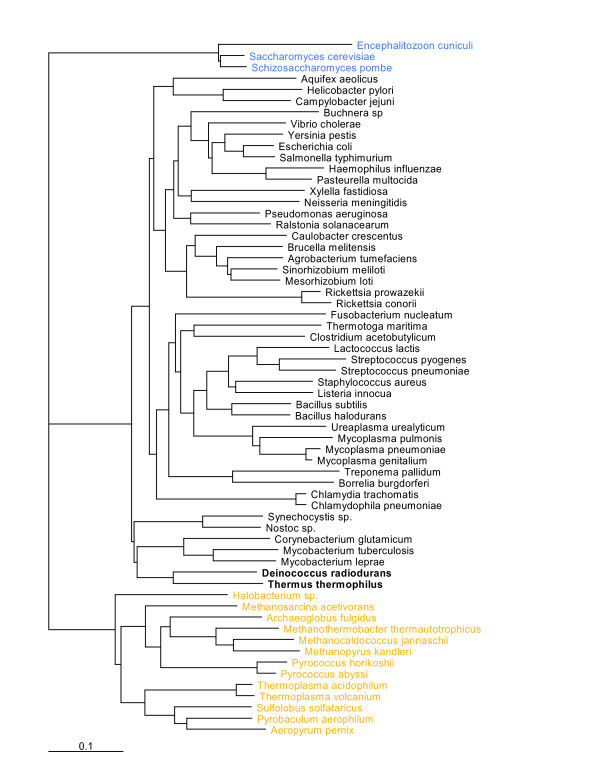
Gene content tree constructed for 66 species included in the COG database on the basis of the patterns of presence-absence in the COGs. The *Thermus-Deinococcus *clade is marked by bold type. Black, bacteria; yellow, archaea; blue, eukaryotes.

**Figure 4:**
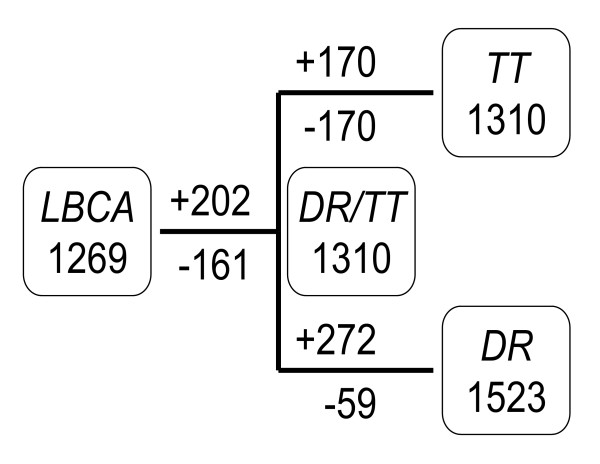
The reconstructed evolutionary scenario for the *Thermus-Deinococcus *clade. LBCA – Last Bacterial Common Ancestor; DR-TT – the common ancestor of *Thermus-Deinococcus *clade; TT – *T. thermophilus*; DR- *D. radiodurans*. Total number of COGs is shown in boxes for each node. '+' indicates inferred gene (COG) gain, and '-' indicates inferred loss.

Information on the presence-absence of orthologous genes in a set of genomes can be used to produce a gene-content tree [33,34]. The topology of a gene-content tree may reflect not only the phylogenetic relationships between the compared species but lifestyle similarities and differences as well [33,35,36]. Given the dramatic differences in the lifestyles and resistance phenotypes of TT and DR, we were interested to determine whether or not the gene content of TT was most similar to that of DR or those of other thermophilic bacteria or, perhaps, even archaea. To this end, we assigned the proteins encoded in the TT genome to the Clusters of Orthologous Groups of proteins (COGs) [37] and, using the patterns of representation of species in COGs to calculate distances between species, reconstructed a gene-content tree as described previously [35]. In the resulting gene-content tree, which included 62 sequenced genomes of prokaryotes and unicellular eukaryotes, TT and DR were confidently recovered as sister species, and the DR-TT lineage was positioned within a subtree that also included Actinobacteria and Cyanobacteria, several of which are known for their extreme radiation and desiccation resistances [38-40] (Figure 3). For this branch, the topology of the gene-content tree mimics the topologies of trees constructed with other approaches based on genome-wide data [34,35], indicating that the gene repertoires of these bacteria, and TT and DR in particular, have been diverging, roughly, in a clock-like fashion. To determine which genes were likely to have been lost and gained in each lineage, we reconstructed a parsimonious scenario of evolution from the last bacterial common ancestor (LBCA) to TT and DR, through their last common ancestor. The reconstruction was performed on the basis of the assignment of TT and DR proteins to COGs, together with COG-based phyletic patterns of 62 other sequenced bacterial and archaeal genomes [37], using a previously developed weighted parsimony method [41] (see Methods and Additional file 2). This approach assigns 1,310 genes (COGs) to the DR-TT common ancestor (Figure 4). Of these, 1,081 (~80%) were retained in both TT and DR and belong to their shared gene core. Since TT (2210 genes) has far fewer predicted protein-coding genes than DR (3191 genes), it seems likely that the divergence of the two involved substantial genome reduction in TT and/or genome expansion in DR. However, the reconstruction results suggest that TT has not experienced massive genome reduction although the total gene flux (i.e., the sum total of genes inferred to have been lost and gained) during the evolution of this lineage was considerable, involving ~25% of the gene complement. In contrast, DR gained 272 COGs, with only 59 lost, which indicates substantial genome growth after the DR-TT divergence (Figure 4).

We were further interested in determining whether similarities existed among the gene repertoires of TT and two deeply-branching bacterial hyperthermophiles, *Aquifex aeolicus *(AA) and *Thermotoga maritima *(TM). We found that genes that are present in TT but not DR are significantly more likely to be present in AA and TM than genes present in DR but not TT (Table 1). In contrast, among the 170 TT genes inferred to have been lost, only 20 were present in both AA and TM. Thus, the gene repertoire of TT has significantly greater similarity with hyperthermophilic bacteria than the gene repertoire of DR, perhaps resulting from direct or parallel HGT (see also below).

**Table 1:** Concordant and discordant phyletic patterns between DR, TT, *Aquifex aeolicus *(AA) and *Thermotoga maritima *(TM)

	**AA+TM+**	**AA-TM-**
**DR-TT+**	47 (21.7)	110 (135.3)
**DR+TT-**	23 (48.3)	326 (300.7)

Note: Expected number of COGs under the assumption of independence is shown in parentheses. Probability, associated with the χ^2 ^test, is 2 × 10^-12 ^for the complete 2 × 2 table and 3 × 10^-6 ^for the 2 × 1 table (concordant vs. discordant).

The majority of the genes shared by TT and DR encode house-keeping proteins and are widespread in bacteria. Among those, 14 COGs are unusual in that they are not found in any other bacteria but instead are shared by TT and DR with archaea or eukaryotes. This set includes 8 subunits of the Archaeal/vacuolar-type H+-ATPase and six other COGs that consist of characteristic archaeal genes (phosphoglycerate mutase, COG3635; 2-phosphoglycerate kinase, COG2074; SAR1-like GTPase, COG1100; GTP:adenosylcobinamide-phosphate guanylyltransferase, COG2266; Predicted membrane protein, COG3374 ; Predicted DNA modification methylase, COG1041).

As noticed previously, some DR genes that belong to families well-represented in both bacteria and archaea showed clear archaeal affinity [42]. To assess how many genes of apparent "thermophilic" descent (either archaeal or bacterial) might already have been present already in the common DR-TT ancestor, we performed phylogenetic analysis of genes that were assigned to the DR-TT ancestor and had 3 of the 5 best hits to the genes from thermophiles (both archaeal and bacterial; see Additional file 1: "Phylogenetic analysis of the genes of the reconstructed DR-TT common ancestor"). We found that at least 122 genes (~10% of the predicted gene repertoire of the DR-TT common ancestor) of the originally selected 205 genes showed an affinity to thermophilic species, i.e., either a branch of two orthologous genes from DR and TT, or a DR or TT gene (in cases when the respective ortholog apparently was lost in the other lineage) clustered with thermophiles (see Additional file 1, table 1S; and Additional file 6). Due to the fact that many tree topologies are highly perturbed by multiple HGT events and may be inaccurate due to differences in evolutionary rates between lineages, this is only a rough estimate of the number of "thermophilic" genes in the DR-TT ancestor. In particular, it cannot be ruled out that some of the ancestral "thermophilic" genes, which are currently present in TT but not DR (10 such genes out of 122 tested genes), have been acquired by TT via XGD (see Additional file 1, table 1S). Taken together, these observations suggest the possibility of ecological contacts between the DR-TT ancestor and hyperthermophilic archaea and/or bacteria, leading to substantial acquisition of "thermophilic" genes via HGT.

### Gene gain and loss in *Thermus*

Our reconstruction of the evolutionary events that occurred after the divergence of the TT and DR lineages from the common ancestor delineated the sets of genes that likely have been gained and lost by each lineage (Figure 4). We first consider in greater detail the pattern of gene loss and gain in TT. The absence of certain metabolic genes in TT creates gaps in its metabolic pathways, some of which are essential. However, TT is capable of synthesizing all amino acids, nucleotides and a majority of cofactors, suggesting that the gaps are filled by analogous or at least non-orthologous enzymes. Several such cases have been described. For example, TT and DR encode unrelated thymidylate synthases, DR2630 (COG0207) and TTC0731 (COG1351). The classical, folate-dependent thymidylate synthase (COG0207) present in DR is probably ancestral in bacteria and apparently was displaced via HGT in the *Thermus *lineage by the flavin-dependent thymidylate synthase, typical of archaea and bacterial thermophiles. In other cases, the substituted analogous enzymes or pathways remain uncharacterized. For instance, the displacement of the folate-dependent thymidylate synthase with the flavin-dependent type in the TT lineage and in other bacteria and archaea correlates with the apparent loss of dihydrofolate reductase (folA, COG0262), which catalyzes the last step of the tetrahydrofolate biosynthesis pathway. Since tetrahydrofolate is an essential cofactor, a displacement appears most likely. Recently, it has been shown that the halobacterial FolP-FolC fusion protein complemented a *Haloferax volcanii folA *mutant [43]. Thus, it appears likely that FolC and FolP in TT and other organisms complement the activity of FolA although the existence of an unrelated, as yet uncharacterized dihydrofolate reductase cannot be ruled out.

In addition, TT does not encode two enzymes for pyridoxal phosphate biosynthesis (pdxK, COG0259 and pdxH, COG2240), while DR has a complete set of enzymes of this pathway. Our reconstructions suggest that *pdxK *was likely lost in the TT lineage, whereas DR probably independently acquired *pdxH*. However, the pathway is likely to be functional in both organisms. Since similar gaps in the pyridoxal phosphate biosynthesis are seen in a variety of prokaryotes [44], it appears that, for at least some steps of this pathway, there exists a set of distinct enzymes which remain unidentified.

Some systems apparently were completely lost in the TT lineage. These include the urease complex, the ramnose metabolism pathway, acyl CoA:acetate/3-ketoacid CoA transferase, fructose transport and utilization, and glycerol metabolism. Notably, most of these systems are also absent in thermophilic bacteria and many thermophilic archaea.

**Figure 7:**
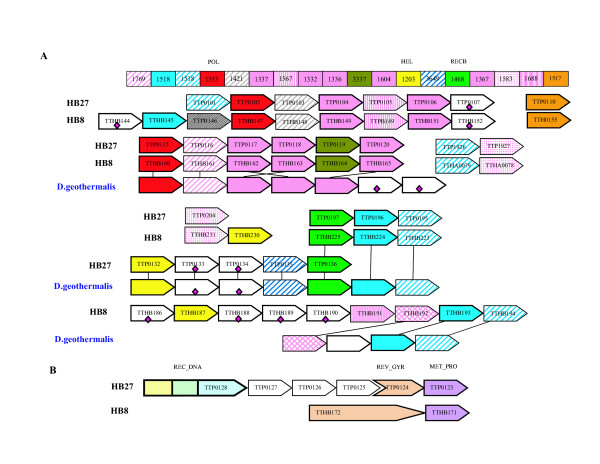
**A. **Organization of genes encoding the putative thermophyle-specific DNA repair system in two *Thermus *strains and the draft genome of *D. geothermalis*. The boxes on top of the figure show COG numbers. Genes are shown by block arrows indicating the direction of transcription and identified by their systematic names. For each column of the alignment, the corresponding COG number and predicted function is indicated. *D. geothermalis' *genes are connected with respective orthologs in both TT strains by a straight line. Generally, orthologous genes are shown by the same color and pattern. The exceptions are the RAMP proteins of COGs 1336, 1367, 1604, 1337 and 1332, which are all shown in pink. Other, more distant RAMPs are also shown in pink, with different patterns [16]. Proteins that do not belong to COGs but have homologs in other species are marked by diamonds. Abbreviations: HEL, predicted helicase, HD nuclease – HD conserved motif containing predicted nuclease conserved region; POL – novel predicted polymerase, RECB – predicted nuclease of RecB family; **B. **Comparison of gene organization in the region of the megaplasmid coding for reverse gyrase in two TT strains. The shorter gyrase gene in HB27 indicates truncation. Abbreviations: REVGYR, reverse gyrase, MET_PR – predicted metal-dependent protease; REC_DNA – three-domain fusion protein (DnaQ endonuclease, DinG helicase, and RecQ helicase).

In contrast to DR, the genes that appear to have been acquired by TT show a clear connection to the thermophilic lifestyle. In particular, TT seems to have acquired 23 gene families from the set of putative thermophilic determinants [17], whereas the common DR-TT ancestor had 5 genes from the list, and DR seems to have acquired only one (see Additional file 3). The majority of these proteins (17 of 31) are encoded in the TT megaplasmid and 11 belong to the predicted mobile DNA repair system characteristic of thermophiles [16,45] (Figure 7A; see details below). In addition, TT has acquired 4 "archaeal" genes that are not encoded in any of the genomes of mesophilic bacteria assigned to COGs (peptide chain release factor 1, COG1503; DNA modification methylase, COG1041; and two membrane proteins, COG3462 and COG4645).

The Sox-like sulfur oxidation system is among the group of genes that were apparently acquired in the TT lineage. The TT Sox operon is partly similar to the one identified in AA (see Additional file 1, Figure 2S), and might have been horizontally transferred between the AA and TT lineages with subsequent local rearrangements. The presence of the Sox operon in TT suggests that this bacterium can use reduced sulfur compounds as a source of energy and sulfur. Another system likely acquired by TT is lactose utilization (at least three proteins: LacZ, COG3250; GalK, COG0153; GalT COG1085; GalA, COG3345). This system is also present in TM, which indicates that sugars can be utilized as carbon sources by thermophilic bacteria.

### Gene gain and loss in *Deinococcus*

The DR lineage has apparently acquired many more genes than it has lost (Figure 4). The majority of the genes lost by DR encode enzymes of energy metabolism and biosynthesis of cofactors. One example is the loss of the three subunits of pyruvate:ferredoxin oxidoreductase, which is one of the several known systems for pyruvate oxidation, a key reaction of central metabolism. Another example of gene loss in DR involves the three subunits of NAD/NADP transhydrogenase, which is responsible for energy-dependent reduction of NADP+ [46]. In addition, the DR lineage lost four enzymes of NAD biosynthesis and six enzymes of cobalamine biosynthesis, and consistently, DR is dependent on an exogenous source of NAD for growth [28,47].

A conspicuous number of genes apparently acquired by the DR lineage encode systems of protein degradation and amino acid catabolism (*e.g*., urease, DRA0311-DRA0319, and a predicted urea transporter, DRA0320-DRA0324; histidine degradation system, DRA0147-DRA0150; monoamine oxidase, DRA0274; lysine 2,3-aminomutase, DRA0027; kynureninase and tryptophan-2,3-dioxygenase, DRA0338-DRA0339; peptidase E, DR1070, and carboxypeptidase C, DR0964; and D-aminopeptidase, DR1843 (see Additional file 4). A similar trend is observed for the expansion of several protein families in DR, such as secreted subtilisin-like proteases (see below). Additionally, DR acquired two three-subunit complexes of aerobic-type carbon monoxide dehydrogenase (DRA0231-DRA0233 and DRA0235-DRA0237); oxidation of CO by this enzyme might be used as an energy source as shown for some bacteria [48]. Acquisition and expansion of these metabolic systems, together with the loss of certain biosynthetic capabilities, supports the possibility that metabolic restructuring could impact oxidative stress resistance in DR by decreasing the need for high-energy-dependent cellular activities. Energy production through the respiratory chain is one major source of free radicals in the cell [49].

DR has many more genes for proteins involved in inorganic ion transport and metabolism than TT. In particular, DR has acquired the multisubunit Na+/H+ antiporter (7 genes), K+-transporting ATPase (3 genes), and the FeoA/FeoB Fe transport system. This abundance of ion transport systems might be indirectly linked to oxidative stress resistance through regulation of membrane ion gradients and Mn/Fe homeostasis (see above).

DR is more dependent than TT on peptide-derived growth substrates [47] and has a more complex stress response circuitry. Consistent with this, the signal transduction systems of DR, as predicted by genome analysis, are considerably more elaborate than those of TT. In particular, DR has at least 33 COGs (15 probably acquired after the divergence from the common ancestor with TT) related to signal-transduction functions that are not represented in TT as compared to 12 (5 acquired) such COGs in TT. Furthermore, although most of the signal-transduction domains are shared by DR and TT, the domain architectures of the respective multidomain proteins are completely different ([7] and KSM, unpublished observations).

Among the genes apparently acquired by DR, two encode multidomain proteins containing distinct periplasmic ligand-binding sensor domains (DRA0202, COG5278 and DR1174, COG3614). Another protein, DRA0204, contains the CHASE3 domain [50] and is located in a predicted operon with superoxide dismutase (DRA0202), indicating a function in oxidative stress response. The protein DR0724 contains the SARP domain which is involved in apoptosis-related signaling pathways in eukaryotes [51] but its function in bacteria is unknown. The roles of the other signal transduction proteins of DR are even less clear, with the notable exception of a phytochrome-like protein (DRA0050) that apparently was acquired by DR from a bacterial source and has been implicated in UV resistance [52].

Additionally, DR has many genes (25 COGs) encoding systems for microbial defense; TT has only 14 COGs in this category, 13 of which are shared with DR. At least 7 genes for restriction-modification system subunits were specifically acquired by the DR lineage, along with several antibiotic-resistance enzymes. This difference might be linked to the reduced metabolic capabilities of DR, which is dependent on nutrient-rich conditions for growth and, perhaps, encounters more microbial species than TT.

The previous analysis of the DR genome revealed 15 genes that appear to have been horizontally transferred from unexpected sources, such as eukaryotes and viruses [7]; only two of these 15 genes are present in TT, the desiccation-related protein of the ferritin family and the Uma2-like family proteins (see discussion of these proteins below). Two desiccation-related proteins have been shown to be involved in desiccation but not radiation resistance [53]; Ro ribonucleoprotein is apparently involved in UV resistance [54], and topoisomerase IB, while active, has no known role in DR [55]. So far, none of these genes has been linked experimentally with radioresistance in DR.

### Identification of xenologous gene displacement by phylogenetic analysis

It is well-established that HGT has made major contributions to the gene repertoires of most thermophilic bacteria as supported by the presence of numerous genes with unexpectedly high similarity to and/or phylogenetic affinity with genes typical of hyperthermophilic archaea [56-60]. These cases include even those proteins that have orthologs in mesophilic bacteria but, as shown by phylogenetic analysis, have clear affinity to archaea or thermophilic bacteria from distant bacterial lineages, which is indicative of XGD [57]. To investigate the impact of HGT from thermophiles leading to XGD on the evolution of the gene repertoire of TT, we determined the taxonomic affiliations of the proteins from the common gene core of TT and DR. We used the taxonomic distribution of best hits in BLAST searches for preliminary identification of HGT candidates, followed by a detailed phylogenetic analysis of selected genes.

**Figure 5:**
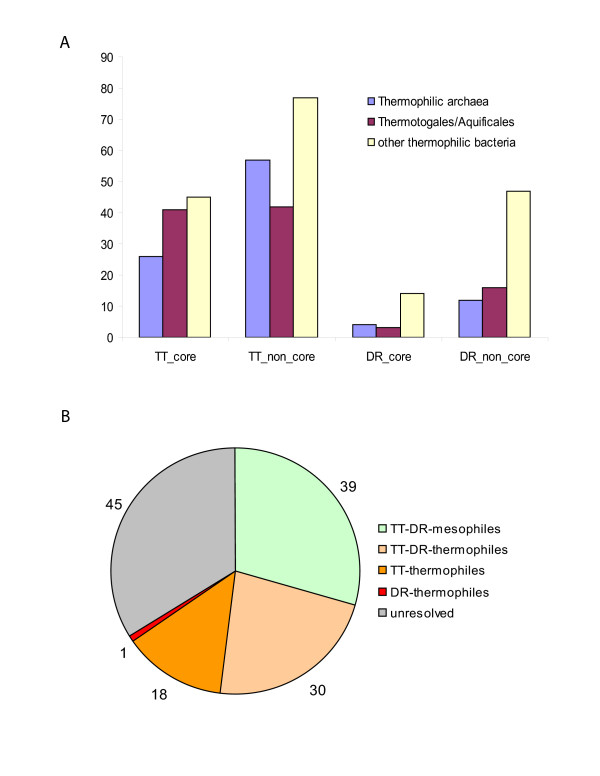
Taxonomic affinities of TT and DR proteins. A. Distribution of the numbers of best hits to proteins from thermophiles for core and non-core proteins of DR and TT. B. Distribution of phylogenetic affinities of proteins from the DR-TT core that have best hit to proteins from thermophiles.

As expected, compared to DR, TT has a notable excess in the fraction of best hits to thermophilic bacteria and archaea for both core and non-core proteins (Figure 5A; and see Additional file 5). However, it has been reported that the best BLAST hit does not always accurately reflect phylogeny [61]. In particular, artifacts of best hit analysis may be caused by similar biases in the amino acid composition of proteins in TT and other thermophiles, as demonstrated previously [62,63]. To assess these effects systematically, we performed phylogenetic analysis of 112 TT proteins and 21 DR proteins from the common core that had their respective best hits in thermophiles (see Additional file 7). Despite the fact that all these trees were built for families in which TT and DR proteins were not mutual best hits in the non-redundant protein sequence database (National Center for Biotechnology Information, NIH, Bethesda), more than half of the trees (69 trees, 52%) recovered a DR-TT clade, 39 of these grouping this clade with mesophiles and 30 with thermophiles (Figure 5B). Nevertheless, the difference of evolutionary patterns of DR and TT came across clearly in this analysis: a reliable affinity with thermophiles was detected for 18 TT proteins and only one DR protein. The former cases are likely to represent HGT into the TT lineage from other thermophiles, whereas the only "thermophilic" gene of DR may involve the reverse direction of HGT, from the DR lineage to *Thermoanaerobacter tencongiensis *(data not shown).

Amino acid composition bias is known to affect not only sequence similarity searches but phylogeny reconstruction as well [64]. We tested this effect on our data set by comparing the sequence-based maximum likelihood trees to the neighbor-joining trees reconstructed from the amino acid frequencies of corresponding proteins (see Additional file 1, "Influence of amino acid composition on phylogenetic reconstructions"). We found that, in the majority of cases (>80%), the topology of the sequence-based tree was not congruent with that of the amino acid composition tree; thus, the effect of the amino acid composition on the breakdown shown in Figure 5B is unlikely to be substantial. Taken together, these results suggest that XGD involving genes from thermophiles made a measurable contribution to the evolution of the core gene set of TT after the divergence from the common DR-TT ancestor; no such contribution was detected in the case of DR. While this interpretation seems most plausible given the ecological proximity of TT and other thermophiles, it cannot be ruled out that the observed patterns (Figure 5B) are partially explained by XGD with genes from mesophilic bacteria in the DR lineage. More generally, these results emphasize that the taxonomic distribution of best database hits can be taken only as a rough and preliminary indicator of HGT.

**Figure 6:**
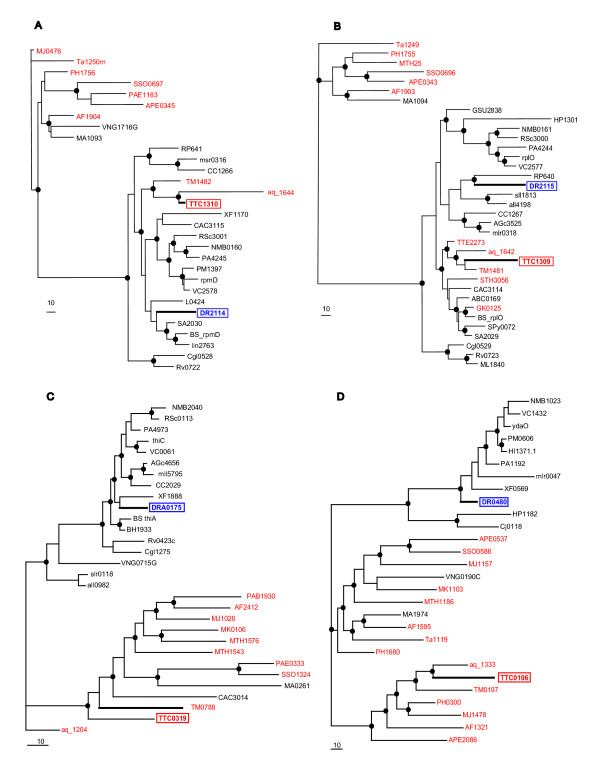
Phylogenetic trees for selected TT genes with apparent XGD involving an ortholog from a thermophilic species. Maximum-likelihood unrooted trees were constructed and bootstrap probabilities were computed using the MOLPHY program. Branches with bootstrap probability >70% are marked by black circles. Each leaf is denoted by the standard gene identifier (for the the complete list of correspondence between genes and species, see Additional file 1: "Gene identifiers and species names for figure 6"). The DR and TT genes are boxed. Genes from thermophilic species are shown in red. A. Ribosomal protein L30. B. Ribosomal protein L15. C. Thiamin biosynthesis protein ThiC. D. tRNA thiolation enzyme TtcA.

Among the cases of potential XGD supported by phylogenetic analysis, there are two ribosomal proteins, L30 and L15, which are encoded by adjacent genes within a conserved ribosomal operon. The proteins encoded by surrounding genes in these operons showed clear affinity to the corresponding DR orthologs (data not shown). In phylogenetic trees of L30 and L15, the TT proteins reliably clustered with orthologs from bacterial hyperthermophiles and not with the corresponding DR orthologs (Figure 6A and 6B). The congruent evolutionary patterns seen with these two ribosomal proteins encoded by adjacent genes suggest that this gene pair has been replaced via XGD *in situ*, without disruption of the operon organization [65]. Additional cases of apparent XGD, where DR confidently partitions into the mesophilic clade, whereas TT belongs to the thermophilic clade, are shown in Figure 6C, D (in each of these cases, the thermophilic clade has an admixture of mesophilic species whereas the mesophilic clade includes no thermophiles).

The apparent lineage-specific HGT in DR and TT was not limited to XGD or to acquisition of genes from thermophiles. Additional examples of various types of HGT supported by phylogenetic analysis are given in Table 2.

**Table 2:** Examples of horizontally transferred genes in TT and DR

**Selected horizontally transferred genes/operons**	***T. thermophilus *Protein ID**	***D. radiodurans *Protein ID**	**Comment**
**Xenologous gene displacement**			
Ribosomal protein L15 (COG0200)	TTC1309	DR2115	Proteobacterial affinity of DR; Affinity to Thermatoga/Aquifex/other Gram-positive bacteria in TT
Ribosomal protein L30/L7E (COG1841)	TTC1310	DR2114	Thermatogales/Aquifecales version in TT, affinity to other bacteria in DR
Thiamine biosynthesis protein ThiC (COG0422)	TTC0319	DRA0175	Proteobacterial affinity in DR; Cyanobacteria/Actinobacterial affinity in TT
Threonine synthase (COG0498)	TTC0117	DRA0360	Proteobacterial version in DR; Cyanobacteria/Actinobacterial affinity in TT
Trk system potassium uptake protein trkG, trkA (COG0168, COG0569)	TT0809, TT0810	DR1667, DR1668; DR1666	Archaeal version in TT; Gram-positive version in DR
Ribonucleoside-diphosphate reductase alpha chain and beta chains (COG0208, COG0209)	TTP0162, TTP0161	DRB0108, DRB0109	Proteobacterial affinity for TT; affinity with Gram-positive bacteria in DR
**Paralogous genes acquisition**			
5-formyltetrahydrofolate cyclo-ligase (COG0212)	TTC1247, TTC1803	DR1815	Gram-positive version in DR and TT; additional pseudoparalog TT1803 appears to be of archaeal origin
**Non-orthologous gene displacement/acquisition**			
Thymidylate synthase	TTC0731 (COG1351)	DR2630 (COG0207)	Represented by two non-homologous genes in DR (protobacterial version) and TT (orthologs in Treponema, Wolbachia)
Purine-nucleoside phosphorylase (purine degradation enzyme)	TTC1070 (COG0813) TTC0194 (COG0005)	DR2166 (COG0813)	TT has two non-homologous enzymes; TTC0194 probably was acquired from a thermopilic source

### Expanded families of paralogs

Most bacterial lineages contain unique sets of expanded paralogous gene families [66]. This notion was borne out by the present comparative-genomic analysis of DR and TT. None of the expanded families that have been detected during the previous detailed analysis of the DR genome [7] was expanded in TT, and many were missing altogether. This strongly suggests that extensive gene duplication and acquisition of new pseudoparalogs via HGT, which led to the expansion of these families in DR, occurred after the divergence from the common ancestor with TT, and could contribute to the specific adaptations of DR (Table 3). Expansion of several other families in DR was revealed in the course of the present comparison with TT. One notable example is the family of predicted membrane-associated proteins (DR2080, DR1043, DR1952, DR1953, DR1738), which are encoded adjacent to transcriptional regulators of the PadR-like family (COG1695; 9 paralogs in DR, none in TT) (Table 3). PadR-like regulators are involved in the regulation of the cellular response to chemical stress agents, derivatives of phenolic acid [67]. Another previously unnoticed paralogous family that is expanded in DR includes proteins containing the MOSC (MOCO sulfurase C-terminal) domain (COG2258, four paralogs in DR and one in TT). These proteins have been predicted to function as sulfur-carriers that deliver sulfur for the formation of sulfur-metal clusters associated with various enzymes [68]. One of these genes (DR0273) forms a predicted operon with genes for a Nudix hydrolase and a monooxygenase, suggesting that these proteins might comprise a distinct stress response/house-cleaning complex.

**Table 3:** Paralogous gene families expanded in DR

**Description**	**COG numbers**	**Number of representatives in DR**	**Number of representatives in TT**
**Widespread families expanded in DR**			
MutT-like phosphohydrolases (Nudix)	COG0494, COG1051	17,5	5,5
Calcineurin-like phosphoesterase	COG1768, COG1408, COG1692, COG0639	1; 1; 1; 9	1; 0; 1; 2
Lipase-like alpha/beta hydrolase	COG0596, COG0400	10; 1	6; 1
Subtilisin-like protease	COG1404	10	2
Sugar deacetylase	COG2120	6	4
PadR-like transcriptional regulators (possibly involved in chemical stress response)	COG1695	9	0
MOSC sulfur-carrier domains	COG2258	4	1
FlaR like kinases	-	3	0
LigT phosphatases (may participate in RNA repair or methabolism)	-	3	2
McrA endonuclease	COG1403	5	1
TerZ family (could confer resistance to a variety of DNA-damaging agents)	COG2310	7	0
PR1 family (stress response)	COG2340	5	1
DinB family (DNA damage and stress inducible proteins)	COG2318; no COG	3; 10	1
Transcriptional regulators	-	5	0
**Unique DR families**			
GRXGG repeats containing protein	**-**	DR0082, DR2593, DR1748	No
Alpha/beta proteins, tryptophan-rich	**-**	DR2532, DR2457	No
Proteins with GXTXXXG and CXPXXXC motifs (DR0871 has duplication of the domain)	**-**	DR0871, DR1920, DR2360	No
Secreted alpha/beta proteins with a single conserved domain	-	DR1251, DR1319, DR1545	No
Predominantly alpha-helical proteins	**-**	DR0481, DR1195, DR1301	No
Predominantly alpha-helical proteins	**-**	DR0387, DR2038+DR2039	No
Predicted metabolic regulator containing V4R domain	-	DR2179, DR1611	No
Predicted sirohydrochlorin cobaltochelatase	COG2138	DRA0012, DR2241	No
Conserved histidine rich protein (now also found in Caulobacter and Mesorhizobium)	COG3798	DR1261, DR1348	No

Several paralogous families are specifically expanded in TT (Table 4). The largest of these (15 paralogs compared to three in DR) is the Uma2 family that is highly expanded in cyanobacteria but otherwise seen in only a few bacteria. The function(s) of these proteins is unknown; the presence of conserved acidic residues suggests that they might be uncharacterized DNA-binding proteins [69]. The expansion of predicted sugar transporters in TT and the paucity of extracellular proteases (including subtilases) is unexpected because it has been shown that TT is predominantly a proteolytic rather than a saccharolytic organism [70]. However, it should be noted that TT, unlike DR, has not been observed to secrete proteases (data not shown).

**Table 4:** Paralogous gene families expanded in TT

**Description**	**COG numbers**	**Number of representatives in DR**	**Number of representatives in TT**
Uncharacterized protein conserved in cyanobacteria, Uma2 homolog	COG4636	3	15
Rhodanese-related sulfurtransferase	COG0607	2	6
ABC-type sugar transport system, periplasmic component	COG1653	1	8
ABC-type sugar transport system, permease component	COG0395	1	6
ABC-type sugar transport systems, ATPase components	COG3839	1	3
ABC-type sugar transport systems, permease components	COG1175	2	6
ABC-type Fe3+ transport system, permease component	COG1178	1	3
Fe2+/Zn2+ uptake regulation proteins	COG0735	1	3
Minimal nucleotidyl transferases	COG1708,COG1669	0,3	3,5
PIN-like nucleases	COG1487, COG3744, COG4113, COG4374 COG1848	3,0 0,1 1	2,2 1,1 3
Antitoxin of toxin-antitoxin stability system	COG4118, COG2161	1,3	3,2
Nucleotide-binding proteins of the UspA family	COG0589	2	4
TRAP-type mannitol/chloroaromatic compound transport system, periplasmic component	COG4663	0	3
TRAP-type mannitol/chloroaromatic compound transport system, small permease component	COG4665	0	2
TRAP-type mannitol/chloroaromatic compound transport system, large permease component	COG4664	0	2
Arabinose efflux permease	COG2814	0	2
Predicted phosphoesterases, related to the Icc protein	COG2129	0	2
HEPN, Nucleotide-binding domain	COG2250	0	2
Aldehyde:ferredoxin oxidoreductase	COG2414	0	3
S-adenosylmethionine decarboxylase	COG1586	0	2
Tfp pilus assembly protein FimT	COG4970	0	2
Tfp pilus assembly protein PilE	COG4968	0	2

Notably, several protein families that are expanded in TT but are absent in DR belong to the set of potential thermophilic determinants (HEPN nucleotide-binding domain; predicted phosphoesterases related to the Icc protein) [17] or are expanded in thermophylic archaea (PIN-like nuclease domain, minimal nucleotidyl transferases, UspA-like nucleotide-binding domain) [71], Table 4). In particular, TT has three paralogs of the archaea-specific tungsten-containing aldehyde ferredoxin oxidoreductase (TTC0012, TTC1834, TTP0122, TTP0212), which is the first occurrenceof this enzyme in thermophilic bacteria. However, these enzymes are present in several mesophilic bacteria, and have various substrate specificities and might be involved in sugar, amino acids or sulfur metabolism [72,73].

### Comparison of DNA repair and stress response systems

Comparative analysis of the well-characterized genetic systems for replication, repair and recombination, and related functions in TT and DR shows that fractions of these genes in the respective genomes are very similar (Table 5; see Additional file 1, Table 2S, 3S). The greatest differences were observed among the proteins associated with direct damage reversal (11 in TT versus 26 in DR), which is due to the extraordinary expansion of the NUDIX (MutT-like) family of hydrolases in DR [7]. It should be noted that the majority of these proteins have other substrates than 7,8-dihydro-8-oxoguanine-triphosphate (or diphosphate), which is cleaved by MutT. Consistently, the majority of the NUDIX proteins appear to be "house cleaning" enzymes rather than *bona fide *components of repair systems [74]. Other notable differences include the apparent loss of the SOS-response transcriptional repressor LexA [12] and another SOS-response protein, endonuclease VII (XseAB) in the TT lineage; these proteins seem to have been lost also by another thermophilic bacterium, AA. In contrast, DR has two LexA paralogs (DRA0344 and DRA0074), but their functions remain unclear. A genetic disruption of DRA0344, the paralog that shows greater similarity to the canonical bacterial LexA protein, does not result in sensitivity to DNA damage or impairment of RecA expression [75]. Photolyase (PhrB) and endonuclease IV (nfo) are among the few DNA repair proteins that probably were acquired by TT after the divergence from the common ancestor with DR. In addition, the catalytic subunit of DNA polymerase III of TT (DnaE, TTC1806) has two inserted inteins, whereas the orthologous DR0507 has none. In general, it seems that the conventional DNA-repair systems of TT and DR are closely related to each other and to the respective systems of other free-living bacteria. Thus, the unique, shared features of these systems do not explain the very large difference observed in resistance between TT and DR species.

**Table 5:** Comparison of general repair pathways in DR and TT

**DNA repair pathways**	**TT**	**DR**
DR – direct damage reversal	11	26
BER – base excision repair	10	15
NER – nucleotide excision repair	9	10
mMM – methylation-dependent mismatch repair	7	6
MM – mismatch repair	-	2
MMY – mutY-dependent repair	1	1
VSP – very short path mismatch repair	2	5
RER – recombinational repair	13	15
SOS repair	5	5
MP – multiple pathways	4	4
Total number (Genome fraction)	62 (**2.8%**)	89 (**2.5%**)

However, a conclusion that there are no important differences between the repair systems of TT and DR might be premature. Recently, several additional proteins of DR have been implicated in DNA or RNA repair, either in direct experiments or on the basis of up-regulation following irradiation, complemented with protein sequence analysis. These putative repair enzymes include DRB0094, an RNA ligase [76] that is strongly up-regulated in response to irradiation [27] and might be involved in an uncharacterized RNA repair process; a predicted double-strand break repair complex specific for recovery after irradiation, which consists of DRB0100, a DNA ligase, DRB0098, a protein containing an HD family phosphatase and polynucleotide kinase domains [7]; DRB0099, a predicted phosphatase of the H2Macro superfamily ([27] and KSM, unpublished observations); a double-stranded DNA-binding protein PprA (DRA0346), which stimulates the DNA end-joining reaction *in vitro *[77]; a predicted DNA single-strand annealing protein DdrA (DR0423) [78]; a regulator of radiation response IrrE (DR0167); a metal-dependent protease fused to a helix-turn-helix domain [79,80]; and the uncharacterized protein DR0070 that has been shown to be essential for full resistance to acute irradiation [27]. Among these poorly characterized (predicted) repair proteins of DR, only DdrA has an ortholog in the TT genome. In general, these putative repair genes are sparsely represented in bacteria, and it appears most unlikely that they were present in the common ancestor of TT and DR; most likely, these genes were acquired by the DR lineage via HGT after the divergence from the common ancestor with TT, and might have contributed to the evolution of the resistance phenotype. However, functional relevance of these genes to radiation resistance remains to be confirmed because most of the corresponding knockout mutants showed only relatively small to moderate decreases in radiation resistance [27,78].

Among the unique (predicted) repair enzymes of TT, the most conspicuous ones are the components of the putative thermophile-specific repair system, which are predominantly encoded on the TT megaplasmid (Figure 7A). The functional features of the proteins encoded in this system (COG1203, a DNA helicase, often fused to a predicted HD-superfamily hydrolase; COG1468, a RecB-family exonuclease; COG1353, predicted polymerase) suggest that they are involved in an as yet uncharacterized DNA repair pathway. It has been hypothesized that this novel gene complex might be functionally analogous to the bacterial-eukaryotic system of translesion, mutagenic repair whose central components are DNA polymerases of the UmuC-DinB-Rad30-Rev1 superfamily, which typically are missing in thermophiles [16].

Comparison of proteins comprising various (predicted) systems involved in stress response reveals a greater number and diversity of such proteins in DR, which has 26 COGs with relevant functions that are not represented in TT compared to 3 such COGs in TT (see Additional file 1, Table 4S). Altogether, there are 147 proteins in DR in this category and 86 in TT, suggesting that some of them are additionally expanded in DR (see the section on "Expanded families").

Enzymatic systems of defense against oxidative stress predicted in TT and DR also show important differences. TT has one Mn-dependent superoxide dismutase (SodA) [81] and one Mn-dependent catalase (with no ortholog in DR), whereas DR encodes three superoxide dismutases (one of which is the ortholog of the SodA gene of TT) and three predicted catalases with no TT orthologs [7]. Additionally, DR has a cytochrome C peroxidase (DRA0301) and a predicted iron-dependent peroxidase (DRA0145), enzymes that are likely to provide protection against toxic peroxides [49]. Orthologs of these enzymes are rare among bacteria, suggesting that the *Deinococcus *lineage acquired them via HGT after the divergence of TT and DR from the common ancestor. Reduction of oxidized methionine residues in proteins is crucial for survival of cells under oxidative stress [82,83]. Consistent with this idea, two peptide methionine sulfoxide reductases (PMSRs), MsrA (DR1849) and MsrB (DR1378), are encoded in the DR genome [84,85], whereas none are present in TT. Interestingly, both PMSRs are also missing in *Aquifex*, *Thermotoga *and most thermophilic archaea, suggesting at least two possibilities: either this type of oxidative damage is rare at high temperatures or the known PMSRs are replaced by uncharacterized analogous enzymes due to the inefficiency of the former at high temperatures.

Oxidative stress defense mechanisms also might include control of Mn and Fe partitioning in the cell [29]. Proteins of the Dps/ferritin family are required for the storage of iron in a non-reactive state, which prevents iron-catalyzed formation of hydroxyl radicals, thus protecting the cell from iron toxicity (Fenton-type chemistry) [86]. Two Dps-related proteins are encoded in the DR genome (DR2263, DRB0092, COG1528), and it has been shown that one of them (DR2263) protects DNA from both hydroxyl radical cleavage and from DNase I-mediated cleavage [87]. Some proteins homologous to DPS can non-specifically bind DNA and therefore are viewed as DNA-specific protectors [88]. Like most thermophiles, TT has no proteins of this family but encodes a ferritin from another family (TTC1122).

Since desiccation also causes oxidative stress, proteins involved in desiccation resistance belong to the general cellular defense category [89]. Desiccation-related proteins from at least two distinct families have been detected in DR [7]. These desiccation resistance protein families (Lea 76 family, DR0105 and DR1172; and Lea14 family, DR1372) are not represented in TT. However, three TT proteins (TTP0170, TTP0166, TTP0169) are homologs of another desiccation resistance protein that was originally characterized in a plant, *Craterostigma plantagineum *[90]; DR also has two proteins of this family, DRB0118 and DRA0258. These proteins are distantly related to COG1633 and belong to the ferritin family of iron storage proteins (KSM, unpublished). Highly conserved homologs of these proteins are also present in thermophilic bacteria and archaea. Two desiccation-related proteins (DR1172 and DRB0118) appear to be essential for desiccation resistance but not for radiation resistance in DR [53].

### Comparison of the genome partitions of TT and DR

Both TT and DR have multipartite genomes. To examine possible evolutionary relationships between the genome partitions of TT and DR, we analyzed the distribution of symmetrical best hits (putative orthologs) in the single extra-chromosomal element of TT, the pTT27 megaplasmid, and the three smaller genome partitions of DR (small chromosome, DR412; megaplasmid, DR177 and plasmid, CP1; Table 6). The results of this analysis show that pTT27 has a highly significant excess of orthologs on DR177, suggesting that these two megaplasmids are homologous, *i.e*., probably evolved from a distinct genome partition of the common DR-TT ancestor. Apparently, however, the genomes of the megaplasmids have undergone extensive rearrangements since the divergence from the common ancestor because no conservation of gene order could be identified (data not shown).

**Table 6:** Homology between the DR and TT megaplasmids

**A. **Number of orthologs of TT proteins, encoded in DR genome partitions				
	**DR chromosome**	**DR412**	**DR177+plasmid CP1**	**P(χ^2^)**

pTT27	65 (84.2)	12 (11.9)	25 (6.0)	**7 × 10**^**-15**^

**B. **Number of orthologs of DR proteins encoded in TT genome partitions				

	**TT chromosome**		**pTT27**	**P(χ^**2**^)**

DR412	89 (87. 9)		9 (10.1)	0.7
DR177	10 (26.9)		20 (3.1)	**3 × 10**^**-24**^

Note: Expected number of orthologs under the assumption of independent distribution is shown in parentheses.

Notably, among the putative thermophilic determinants of TT, ~50% are encoded on the megaplasmid (18 out of 36). Of these, 11 belong to the putative mobile thermophile-specific DNA repair system [16,45] (Figure 7A). Additionally, the megaplasmid carries at least four other genes associated with this system, which have not yet been assigned to COGs (Figure 7A). Moreover, the TT megaplasmid also carries a pseudogene for reverse gyrase, the most conspicuous signature protein of hyperthermophiles [15,17].

Recently, the genome of another strain of TT (HB8) has been completely sequenced and became available in public databases [13]. A preliminary comparison of the two TT strains (HB27 and HB8) revealed considerable differences in the gene orders and contents of the megaplasmids (see Additional file 1, Figure 3S). Interestingly, these differences in gene content are derived mostly from genes that appear to be associated with the thermophylic lifestyle. In particular, strain HB8 encodes an intact reverse gyrase. Thus, it appears most likely that the gene for reverse gyrase was acquired from a hyperthermophilic source by the TT lineage and was present in the common ancestor of HB27 and HB8 but decayed in the former. Conversely, HB27 encodes a unique, three-domain fusion protein (DnaQ endonuclease, DinG helicase and RecQ helicase), whereas HB8 lacks the DinG and RecQ orthologs (Figure 7B). Furthermore, there are unexpected differences in the organization of predicted thermophile-specific repair systems between the two strains of TT. Specifically, HB27 contains a "gram-positive version" (TTP0132-TTP0136), whereas HB8 has a "proteobacterial version" of these genes (TTHB186-TTHB194) (Figure 7A).

Furthermore, a nearly complete draft genome sequence of *Deinococcus geothermalis *(DG) has recently become publicly available [91]. Since DG is closely related to DR but is moderately thermophilic, we searched for "thermophilic" genes in DG genome. Using the "thermophilic" protein sequences ([17] and see above) of TT and DR as queries, we identified orthologs of 5 of the 6 DR proteins from this set (four of these are also present in both TT strains) and orthologs of 7 of the remaining 23 "thermophilic" proteins of TT (all components of the predicted thermophilic DNA repair system). These 7 proteins had the respective TT proteins as the best hits, and their monophyly was supported by phylogenetic analysis (data not shown), suggesting that these genes were already present in the genome of the DR-TT common ancestor.

These observations give rise to two hypotheses: (i) the TT megaplasmid is essential for the survival of the organism at high temperatures. Consistent with this idea, we detected an expansion of a two-component toxin-antitoxin system, which consists of a PIN-like nuclease (toxin) and a MazE family transcriptional regulator (antitoxin) [69]. Such a system is known to be responsible for the segregational stability of antibiotic resistance plasmids and other plasmids via selective elimination of cells that have failed to acquire a plasmid copy [92], and/or exclusion of competing plasmids [93]; and (ii) the TT megaplasmid is a dynamic genome compartment and a veritable sink for horizontally transferred genes, some of which might affect the thermophilic phenotype of this bacterium. This is compatible with the considerable differences in gene content observed between the two TT strains.

Specific roles of plasmid-borne genes in recovery from DNA damage have been proposed previously, including class Ib ribonucleotide reductase, periplasmic alkaline phosphatase, and extracellular nuclease, and subsequent analyses revealed at least five other genes implicated in this process (two operons, DRB0098-DRB0100, DRB0094-DRB0084, see above in "Comparison of DNA repair and stress response systems") [27,76]. There is also a toxin-antitoxin system operon in the DR177 megaplasmid (DRB0012a and another gene located immediately upstream of DRB0012a and currently absent from the genome annotation). Additionally, there are five other toxin-antitoxin systems encoded on DR412, the smaller chromosome of DR, which might be related to maintenance of DR177 megaplasmid in DR. The DR412 chromosome appears to have some special features as well. Numerous genes that apparently have been acquired by DR via HGT after the divergence from the common ancestor with TT and are implicated in various processes of amino acid and nucleotide degradation, map to this genome partition. Thus, the megaplasmids of TT and DR (and the smaller chromosome of DR, DR412) appear to have participated in extensive HGT, which might have been important for the evolution of thermophily and radioresistance, although the repertoires of the respective acquired genes are completely different.

## Conclusion

TT and DR share a large core of genes and form a clade in the gene-content tree, which supports the idea that these bacteria form a distinct clade, as indicated previously by phylogenetic analysis of rRNA and various proteins, and that the evolution of their gene complements was, roughly, clock-like. However, major differences between the gene repertoires of TT and DR were observed, indicating that both genomes lost numerous ancestral genes and acquired distinct sets of new genes primarily via HGT. In addition, numerous lineage-specific expansions of paralogous gene families were identified, particularly, in DR.

Some of the differences in the gene repertoires of TT and DR can be linked to the distinctive adaptive strategies of these bacteria. For example, TT appears to have acquired many genes from (hyper)thermophilic bacteria and archaea, whereas DR apparently acquired various genes involved in oxidative stress response and other "house-cleaning" functions from diverse bacterial sources.

The gene content of the TT megaplasmid (pTT27) and the DR megaplasmid (DR177) are sufficiently similar to conclude that they evolved from a common ancestor. To our knowledge, this is the first evidence of persistence of a megaplasmid beyond the genus level. However, the TT megaplasmid also carries many genes whose functions are implicated in the thermophylic phenotype; in particular, components of the predicted thermophile-specific repair system. These megaplasmids are likely to be essential for the survival of both TT and DR, with their maintenance controlled by toxin-antitoxin systems. Furthermore, the substantial differences between the gene repertoires of the megaplasmids of TT strains HB27 and HB8 indicate that this genome partition has been highly dynamic, with high rates of gene loss and HGT events occurring during evolution.

The evolutionary reconstruction based on the parsimony principle is generally compatible with the idea that the common ancestor of TT and DR was a mesophilic bacterium, whereas the thermophylic phenotype of TT evolved gradually via HGT of genes from thermophiles. Conversely, the radiation-desiccation resistance phenotype of DR might have gradually evolved via HGT of genes from other mesophiles, particularly, with highly developed oxidative stress response systems. However, it should be noted that the TT-DR common gene core includes dozens of genes of apparent archaeal origin or, at least, genes with thermophilic affiliation. Moreover, the DG genome encodes a few additional "thermophilic determinants" that are missing in DR but are unlikely to have been transferred from a thermophilic source independently of TT, as shown by comparative-genomic and phylogenetic analyses described here. Thus, acquisition of a considerable number of archaeal genes might have occurred along the evolutionary branch leading to the common ancestor of TT and DR. Accordingly, at this stage, we cannot rule out the possibility that this ancestor was a moderate thermophile rather than a mesophile. Further sequencing of bacterial genomes of the *Thermus-Deinococcus *clade should allow more definitive comparative-genomic analysis to elucidate the nature of the common ancestor of these bacteria.

## Methods

### Irradiation and desiccation

Irradiations. Three TT colony-isolates were inoculated individually in liquid TGY (10 g/L Bactotryptone, 1 g/L glucose, 5 g/L yeast extract) and incubated at 70°C. Cells were harvested at OD_600 _~0.9, which corresponds to 10^7 ^– 10^8 ^colony forming units (CFU)/ml; 1 TT cell/CFU. TT cells grown in TGY were examined for their total Mn and Fe content by ICP-MS (see main text). For radiation resistance assays, cells were irradiated without change of broth on ice with ^60^Co at 6.8 kGy/hour (^60^Co Gammacell irradiation unit [J. L. Shepard and Associates, Model 109]). At the indicated doses, cultures (3 biological replicates) were appropriately diluted and plated on solid medium (8 g/L Bactotryptone, 4 g/L yeast extract, 3 g/L NaCl, pH 7.3, 2.8% Bactoagar), and CFU counts were determined after 2 days' incubation at 65°C.

Desiccation. Five separate colony-isolates were pre-grown in TGY as for irradiation trials. Cell samples 10^6^–10^7 ^cells (25 μl) were transferred to microtiter plates, which were transferred to desiccation chambers containing anhydrous calcium sulfate (drierite) and incubated at room temperature or 65°C. At the indicated times, cells were re-suspended in TGY, and CFU-survival frequencies were determined by dilution-plating on solid medium (65°C).

### Genome analysis

The sets of predicted proteins of TT and DR were searched against each other for symmetrical and non-symmetrical hits using PSI-BLAST with expectation (E) value threshold of 10^-5^. Taxonomic affiliation was determined by best hits in non-redundant database of protein sequences at the National Center of Biotechnology Information (NIH, Bethesda) using BLASTP program [94] with default expectation value (0.01). Assignments to COGs were performed using the COGNITOR program [95] and CDD-search against COG-based profiles [96]. Contradictory assignments were resolved manually. Lineage-specific expansions (LSE) were identified as described previously [66,97]. The common genomic core was determined as follows: among genes that were not assigned to any COG, orthology relationships between TT and DR were determined via symmetrical best hits. Genes belonging to COGs, shared between TT and DR and having only one ortholog from each of the two genomes were directly assigned to the core. For multiple-paralog COGs, symmetrical best hits between GOG members were used to refine the relationships between TT and DR proteins. Members of the corresponding lineage-specific expansions were added to SymBeT pairs to form many-to-many core clusters. LBCA gene set was determined using an empirical parsimony procedure based on COG phyletic patterns (See Additional file 1: "Reconstruction of the gene set of the Last Bacterial Common Ancestor") which assigned a COG to LBCA if it was present in several diverse bacterial clades. All COGs that were present in LBCA and in TT and/or DR were assigned to the DR-TT ancestor.

### Phylogenetic analysis

Multiple alignments for phylogenetic analysis were constructed using the MUSCLE program [98]; columns containing gaps in >30% of the sequences were discarded. Maximum likelihood trees were constructed using the ProtML program of the MOLPHY package by optimizing the least-square trees with local rearrangements [99]. Trees based on amino acid content were constructed from the matrix of Euclidean distances between frequency vectors using the NEIGHBOR program of the PHYLIP package [100]. Support for particular arrangements of species (relationships between DR, TT, thermophiles and mesophiles) was calculated using the bipartition analysis of bootstrap samples from original sequences (see Additional file 1, "Influence of amino acid composition on phylogenetic reconstructions"). The gene-content tree based on COG patterns was constructed using the NEIGHBOR program of the PHYLIP package [100]; the number of COGs shared between two genomes was normalized by the smaller genome size [33].

## Authors' contributions

MVO performed the majority of genomic comparisons, phylogenetic analysis and helped to draft the manuscript. YIW and KSM performed some genomic comparisons and statistical analysis of the data. EKG, VYM, AV and MZ performed the experiments on the comparison of the *Thermus *and *Deinococcus *response to irradiation and desiccation and the analysis of Mn/Fe ratio of *Thermus*. MJD, EVK, and KSM participated in design of experiments and project coordination, and contributed to the writing of the manuscript. All authors read and approved the final manuscript.

## Supplementary Material

Additional File 1 (contains supplementary text and supplementary **tables 1S, 2S, 3S, 4S **and supplementary **figures 1S, 2S, 3S**) Click here for file

Additional File 2 contains the assignments of DR and TT proteins to the COG database. Click here for file

Additional File 6 contains compressed file for all the trees (in PHYLIP format) for 122 proteins from the reconstructed gene set of the DR-TT common ancestor that showed affinity to thermophiles (see also Additional file 1: **"Phylogenetic analysis of the genes of the reconstructed DR-TT common ancestor"**). Click here for file

Additional File 3 contains DR and TT protein comparison with COGs predicted to be associated with the thermophilic phenotype. Click here for file

Additional File 4 contains the results of the reconstruction of the gene repertoire of the common ancestor of TT and DR. Click here for file

Additional File 5 contains information on taxonomic assignments for the best hits of the TT and DR proteins. Click here for file

Additional File 7 contains compressed file for all the trees (in PHYLIP format) for proteins from DR-TT core that had best hits to thermophiles. Click here for file
