# Supplementary material for: Comparative genomics of Thermus thermophilus and Deinococcus radiodurans: divergent routes of adaptation to thermophily and radiation resistance
Source: BMC Evol Biol. 2005 Oct 20;5:57. doi: 10.1186/1471-2148-5-57 (PMC1274311; doi:10.1186/1471-2148-5-57)
Supplement: Additional File 1 — (contains supplementary text and supplementary tables 1S, 2S, 3S, 4S and supplementary figures 1S, 2S, 3S) [file 1471-2148-5-57-S1.doc]

**Desiccation of TT at 65^o^C**

Our standard drying procedure in the presence of anhydrous calcium sulfate (drierite) at room temperature results in complete evaporation of cultures in 6 hours, with humidity levels in chambers falling to ≤14%. At 65^o^C, the rate of desiccation in the presence of drierite is significantly higher. To avoid sudden drying of TT at its optimal growth temperature of 65^o^C, we allowed TT cells to desiccate slowly over a period of 2 days. This approach provided the cells with a physiologic environment favoring gene expression. Slow desiccation at 65^o^C was achieved by incubating 96-well microtiter plates containing TT cells in a covered (unsealed) chamber with sufficient liquid water to sustain 100% humidity levels for at least 30 hours, leading to desiccation of the TT samples at 40 hours. One day later, the desiccated TT samples showed 100% lethality. In contrast, TT cells desiccated at 25^o^C for 6 hours were more resistant, showing 0.0013% survival after 1 day and 100% lethality after 5 days (**Figure 2**). Notably, incubation of TT at 25^o^C did not affect viability, with no loss in CFU survival frequency observed after 5 days (data not shown).

**Phylogenetic analysis of the genes projected to the TT-DR common ancestor.**

All TT and DR proteins from 1310 COGs that were projected to the TT-DR common ancestor were used as queries in a BLAST search against the COG database. If a query had at least 3 of the 5 top BLAST hits to proteins from thermophilic species, the corresponding COG was subjected to further analysis. Some COGs consist of numerous genes (including paralogs), which precludes accurate phylogenetic analysis of the entire sequence set; therefore pre-selection of sequences within a COG is required. We used BLAST search against the COG members with TT and DR proteins as queries and selected up to 20 top-ranking proteins (taking the highest rank among all searches). Multiple alignments were constructed with MUSCLE program {Edgar, 2004 #161}; columns containing >30% of gaps were excluded. Maximum likelihood trees were constructed using the ProtML program of the MOLPHY package by optimizing the least-square trees with local rearrangements {Hasegawa, 1991 #73}. To determine the taxonomic affinity of DR and TT genes, trees were inspected visually on the case-by-case basis. If a COG contained representatives from both TT and DR, which were located on the same branch and clustered with thermophiles, an ancestral HGT from a thermophilic source was inferred; in all other cases, the possibility of this route of HGT was rejected. If a COG included a representative from only one of the analyzed genomes (either DR or TT), an ancestral HGT from thermophiles was inferred only if all paralogs represented in the tree clustered with thermophiles.

Table 1S. Genes of “thermophilic origin” in the reconstructed gene set of the common ancestor of DR and TT

| COG | Group**^A^** | Annotation | Gene | GI number | State**^B^** |
| --- | --- | --- | --- | --- | --- |
| COG0022 | C | Pyruvate/2-oxoglutarate dehydrogenase complex, dehydrogenase (E1) component, eukaryotic type, beta subunit | AcoB | 6457689, 46200058 | LBCA |
| COG0636 | C | F0F1-type ATP synthase, subunit c/Archaeal/vacuolar-type H+-ATPase, subunit K | AtpE | 6458401, 46199215 | LBCA |
| COG0674 | C | Pyruvate:ferredoxin oxidoreductase and related 2-oxoacid:ferredoxin oxidoreductases, alpha subunit | PorA | 46199893 | DRloss |
| COG0843 | C | Heme/copper-type cytochrome/quinol oxidases, subunit 1 | CyoB | 46199076 | LBCA |
| COG1013 | C | Pyruvate:ferredoxin oxidoreductase and related 2-oxoacid:ferredoxin oxidoreductases, beta subunit | PorB | 46199894 | DRloss |
| COG1014 | C | Pyruvate:ferredoxin oxidoreductase and related 2-oxoacid:ferredoxin oxidoreductases, gamma subunit | PorG | 46199893 | DRloss |
| COG1032 | C | Fe-S oxidoreductase | - | 6460688, 6460970, 46199246 | LBCA |
| COG1145 | C | Ferredoxin | NapF | 6459648, 46198330 | LBCA |
| COG1156 | C | Archaeal/vacuolar-type H+-ATPase subunit B | NtpB | 6458406, 46199210 | DRTTgain |
| COG1249 | C | Pyruvate/2-oxoglutarate dehydrogenase complex, dihydrolipoamide dehydrogenase (E3) component, and related enzymes | Lpd | 46199095 | LBCA |
| COG1254 | C | Acylphosphatases | AcyP | 6458652, 46198507 | LBCA |
| COG1436 | C | Archaeal/vacuolar-type H+-ATPase subunit F | NtpG | 46199212 | DRTTgain |
| COG1527 | C | Archaeal/vacuolar-type H+-ATPase subunit C | NtpC | 6458403,46199213 | DRTTgain |
| COG1622 | C | Heme/copper-type cytochrome/quinol oxidases, subunit 2 | CyoA | 46199075 | LBCA |
| COG1052 | CHR | Lactate dehydrogenase and related dehydrogenases | LdhA | 46198739 | DRloss |
| COG0002 | E | Acetylglutamate semialdehyde dehydrogenase | ArgC | 6458685,46199142,46199844 | LBCA |
| COG0006 | E | Xaa-Pro aminopeptidase | PepP | 6458166,46199575 | LBCA |
| COG0010 | E | Arginase/agmatinase/formimionoglutamate hydrolase, arginase family | SpeB | 46199070 | LBCA |
| COG0065 | E | 3-isopropylmalate dehydratase large subunit | LeuC | 6459378,6459551,46199849 | LBCA |
| COG0066 | E | 3-isopropylmalate dehydratase small subunit | LeuD | 6459381,6459552,46199848 | LBCA |
| COG0118 | E | Glutamine amidotransferase | HisH | 46198370 | LBCA |
| COG0119 | E | Isopropylmalate/homocitrate/citramalate synthases | LeuA | 46199151,46199153 | LBCA |
| COG0160 | E | 4-aminobutyrate aminotransferase and related aminotransferases | GabT | 6460573,46198818 | LBCA |
| COG0421 | E | Spermidine synthase | SpeE | 46198780 | LBCA |
| COG0548 | E | Acetylglutamate kinase | ArgB | 6459171,46199843,46199888 | LBCA |
| COG0624 | E | Acetylornithine deacetylase/Succinyl-diaminopimelate desuccinylase and related deacylases | ArgE | 6459166,46199698,46199992 | LBCA |
| COG0665 | E | Glycine/D-amino acid oxidases (deaminating) | DadA | 46199475 | LBCA |
| COG1506 | E | Dipeptidyl aminopeptidases/acylaminoacyl-peptidases | DAP2 | 46199543,6457834 | DRTTgain |
| COG2876 | E | 3-deoxy-D-arabino-heptulosonate 7-phosphate (DAHP) synthase | AroA | 46198756 | LBCA |
| COG3842 | E | ABC-type spermidine/putrescine transport systems, ATPase components | PotA | 46198671,46199361 | LBCA |
| COG4992 | E | Ornithine/acetylornithine aminotransferase | ArgD | 6458508,46199695 | LBCA |
| COG0601 | EP | ABC-type dipeptide/oligopeptide/nickel transport systems, permease components | DppB | 6458040 | LBCA |
| COG1063 | ER | Threonine dehydrogenase and related Zn-dependent dehydrogenases | Tdh | 46198719 | LBCA |
| COG0504 | F | CTP synthase (UTP-ammonia lyase) | PyrG | 6459338,46199404 | LBCA |
| COG0563 | F | Adenylate kinase and related kinases | Adk | 46199609 | LBCA |
| COG0737 | F | 5'-nucleotidase/2',3'-cyclic phosphodiesterase and related esterases | UshA | 46199354 | LBCA |
| COG1828 | F | Phosphoribosylformylglycinamidine (FGAM) synthase, PurS component | PurS | 46199454 | LBCA |
| COG0120 | G | Ribose 5-phosphate isomerase | RpiA | 6458564 | LBCA |
| COG0235 | G | Ribulose-5-phosphate 4-epimerase and related epimerases and aldolases | AraD | 46199761 | DRloss |
| COG1082 | G | Sugar phosphate isomerases/epimerases | IolE | 46197949,46199873 | DRloss |
| COG1653 | G | ABC-type sugar transport system, periplasmic component | UgpB | 46198923 | LBCA |
| COG2074 | G | 2-phosphoglycerate kinase | - | 6459277,46198429 | DRTTgain |
| COG2182 | G | Maltose-binding periplasmic proteins/domains | MalE | 46199590 | DRTTgain |
| COG3635 | G | Predicted phosphoglycerate mutase, AP superfamily | - | 6460031,46200190 | DRTTgain |
| COG0477 | GEPR | Permeases of the major facilitator superfamily | ProP | 46199078,46200085,6457962 | LBCA |
| COG0697 | GER | Permeases of the drug/metabolite transporter (DMT) superfamily | RhaT | 46199599,46200083,46200289 | LBCA |
| COG0007 | H | Uroporphyrinogen-III methylase | CysG | 46198616 | LBCA |
| COG0142 | H | Geranylgeranyl pyrophosphate synthase | IspA | 6459152,46200288 | LBCA |
| COG0156 | H | 7-keto-8-aminopelargonate synthetase and related enzymes | BioF | 6460157,46199521 | LBCA |
| COG0212 | H | 5-formyltetrahydrofolate cyclo-ligase | - | 46200105 | LBCA |
| COG0611 | H | Thiamine monophosphate kinase | ThiL | 46198364 | DRloss |
| COG0746 | H | Molybdopterin-guanine dinucleotide biosynthesis protein A | MobA | 46199241 | LBCA |
| COG1270 | H | Cobalamin biosynthesis protein CobD/CbiB | CbiB | 46197939,6460868 | LBCA |
| COG1541 | H | Coenzyme F390 synthetase | PaaK | 46198443 | DRTTgain |
| COG1977 | H | Molybdopterin converting factor, small subunit | MoaD | 46200137 | LBCA |
| COG2104 | H | Sulfur transfer protein involved in thiamine biosynthesis | ThiS | 46198413 | LBCA |
| COG2243 | H | Precorrin-2 methylase | CobF | 46197931 | DRloss |
| COG0111 | HE | Phosphoglycerate dehydrogenase and related dehydrogenases | SerA | 46198894,6459038 | LBCA |
| COG0189 | HJ | Glutathione synthase/Ribosomal protein S6 modification enzyme (glutaminyl transferase) | RimK | 6459994,46199845 | LBCA |
| COG0020 | I | Undecaprenyl pyrophosphate synthase | UppS | 6460263,46199853 | LBCA |
| COG0183 | I | Acetyl-CoA acetyltransferase | PaaJ | 46198499 | LBCA |
| COG0365 | I | Acyl-coenzyme A synthetases/AMP-(fatty) acid ligases | Acs | 46199189,46199223,6458145 | LBCA |
| COG1250 | I | 3-hydroxyacyl-CoA dehydrogenase | FadB | 6460513 | LBCA |
| COG1884 | I | Methylmalonyl-CoA mutase, N-terminal domain/subunit | Sbm | 6458931,46198985,46199186 | DRTTgain |
| COG1960 | I | Acyl-CoA dehydrogenases | CaiA | 46197994 | LBCA |
| COG2185 | I | Methylmalonyl-CoA mutase, C-terminal domain/subunit (cobalamin-binding) | Sbm | 6459815,46198984 | DRTTgain |
| COG0017 | J | Aspartyl/asparaginyl-tRNA synthetases | AsnS | 6458780,46199389 | LBCA |
| COG0018 | J | Arginyl-tRNA synthetase | ArgS | 6460396,46200208 | LBCA |
| COG0172 | J | Seryl-tRNA synthetase | SerS | 6459024,46198828 | LBCA |
| COG0480 | J | Translation elongation factors (GTPases) | FusA | 6458071 | LBCA |
| COG1187 | J | 16S rRNA uridine-516 pseudouridylate synthase and related pseudouridylate synthases | RsuA | 46200149 | LBCA |
| COG0846 | K | NAD-dependent protein deacetylases, SIR2 family | SIR2 | 46199328 | LBCA |
| COG1522 | K | Transcriptional regulators | Lrp | 6460096,46198801 | LBCA |
| COG0177 | L | Predicted EndoIII-related endonuclease | Nth | 6458651,6460251 | LBCA |
| COG0433 | L | HerA helicase | - | 6458555,46198455,46200181 | DRTTgain |
| COG0582 | L | Integrase | XerC | 6460964 | LBCA |
| COG0863 | L | DNA modification methylase | - | 46198349,46200182 | LBCA |
| COG1796 | L | DNA polymerase IV (family X) | POL4 | 46199091 | DRTTgain |
| COG1943 | L | Transposase | - | 6457846,6458366 | TTloss |
| COG0381 | M | UDP-N-acetylglucosamine 2-epimerase | WecB | 46198593 | LBCA |
| COG0449 | M | Glucosamine 6-phosphate synthetase, contains amidotransferase and phosphosugar isomerase domains | GlmS | 6457976 | LBCA |
| COG0463 | M | Glycosyltransferases involved in cell wall biogenesis | WcaA | 46198810,6458811 | LBCA |
| COG1088 | M | dTDP-D-glucose 4,6-dehydratase | RfbB | 6460562 | TTloss |
| COG1091 | M | dTDP-4-dehydrorhamnose reductase | RfbD | 6460559 | TTloss |
| COG1209 | M | dTDP-glucose pyrophosphorylase | RfbA | 6460571,46200064 | LBCA |
| COG5479 | M | Uncharacterized protein potentially involved in peptidoglycan biosynthesis | - | 46199709 | DRTTgain |
| COG2804 | NU | Type II secretory pathway, ATPase PulE/Tfp pilus assembly pathway, ATPase PilB | PulE | 6459749,46199924,46200146 | LBCA |
| COG0755 | O | ABC-type transport system involved in cytochrome c biogenesis, permease component | CcmC | 46199443 | LBCA |
| COG0526 | OC | Thiol-disulfide isomerase and thioredoxins | TrxA | 46198794 | LBCA |
| COG0616 | OU | Periplasmic serine proteases (ClpP class) | SppA | 46199413 | LBCA |
| COG1055 | P | Na+/H+ antiporter NhaD and related arsenite permeases | ArsB | 46199749 | DRloss |
| COG1117 | P | ABC-type phosphate transport system, ATPase component | PstB | 46200025 | LBCA |
| COG1324 | P | Uncharacterized protein involved in tolerance to divalent cations | CutA | 6460102,46199293 | DRTTgain |
| COG2217 | P | Cation transport ATPase | ZntA | 46199673 | LBCA |
| COG1335 | Q | Amidases related to nicotinamidase | PncA | 46199957 | LBCA |
| COG0110 | R | Acetyltransferase (isoleucine patch superfamily) | WbbJ | 46198594 | LBCA |
| COG0446 | R | Uncharacterized NAD(FAD)-dependent dehydrogenases | HcaD | 46199349,46199701,46199786 | LBCA |
| COG0491 | R | Zn-dependent hydrolases, including glyoxylases | GloB | 6459186,46198766,6460803 | LBCA |
| COG0535 | R | Predicted Fe-S oxidoreductases | - | 6460969 | TTloss |
| COG1011 | R | Predicted hydrolase (HAD superfamily) | - | 6458094,46199447,46199456 | LBCA |
| COG1092 | R | Predicted SAM-dependent methyltransferases | - | 6459463,46199218,46199431 | LBCA |
| COG1100 | R | GTPase SAR1 and related small G proteins | - | 6458569,46199073 | DRTTgain |
| COG1547 | R | Metal-dependent hydrolase, possible protease | - | 6458676 | DRTTgain |
| COG1832 | R | Predicted CoA-binding protein | - | 6458247 | DRTTgain |
| COG1853 | R | Conserved protein/domain typically associated with flavoprotein oxygenases, DIM6/NTAB family | - | 46197970,46199809 | LBCA |
| COG2018 | R | Uncharacterized distant relative of homeotic protein bithoraxoid | - | 6458313,6459379,6459980 | DRTTgain |
| COG2151 | R | Predicted metal-sulfur cluster biosynthetic enzyme | PaaD | 46198451 | DRTTgain |
| COG2872 | R | Predicted metal-dependent hydrolases related to alanyl-tRNA synthetase HxxxH domain | - | 6458192,46200150 | DRTTgain |
| COG3294 | R | HD supefamily hydrolase | - | 6459204,46199370 | DRTTgain |
| COG3375 | R | NH2-acetyltransferase | - | 6457705,6460941,46198826 | DRTTgain |
| COG0327 | S | Uncharacterized conserved protein, NIF3 homologs | - | 6457771,46199544 | LBCA |
| COG1690 | S | Uncharacterized conserved protein | RtcB | 46199728 | DRTTgain |
| COG1917 | S | Sugar phosphate isomerase family enzyme | - | 46199399 | DRloss |
| COG2120 | S | Uncharacterized proteins, LmbE homologs | - | 6460546,46199145 | DRTTgain |
| COG2383 | S | Uncharacterized conserved membrane protein | - | 6457864,46199742 | DRTTgain |
| COG3374 | S | Predicted membrane protein | - | 46199564 | DRTTgain |
| COG0589 | T | Universal stress protein UspA and related nucleotide-binding proteins | UspA | 46198847 | LBCA |
| COG2206 | T | HD-GYP domain | - | 6459329,46197999,46198087 | DRTTgain |
| COG0577 | V | ABC-type antimicrobial peptide transport system, permease component | SalY | 6458160,46199784 | LBCA |
| COG0842 | V | ABC-type multidrug transport system, permease component | - | 46198882,46198883 | LBCA |
| COG1131 | V | ABC-type multidrug transport system, ATPase component | CcmA | 46198881,46199397 | LBCA |
| COG1132 | V | ABC-type multidrug transport system, ATPase and permease components | MdlB | 46199278 | LBCA |

Note:

**^A^** Functional group according to COGs (http://www.ncbi.nlm.nih.gov/COG/old/palox.cgi?fun=all)

**^B^** State of the gene according to reconstruction of gene loss and gene gain events, starting from LBCA (last bacterial common ancestor). Abbreviations are the following: LBCA – gene came from last bacterial common ancestor; DRTTgain – gene gained by DR/TT common ancestor; DRloss – gene present in DR/TT common ancestor, but lost in DR lineage; TTloss – gene present in DR/TT common ancestor, but lost in TT lineage;

TTC1057

TTC1056

TTC1055

TTC1054

TTC1053

TTC1052

TTC1051

TTC1050

TTC1049

TTC1048

TTC1047

TTC1046

COG 0446

COG2041,

soxC

COG3439

COG5501,

soxY

COG0526

tioredoxin

COG0737,

soxB

soxZ

_2

DRA0018

DRA0225

soxA

,

cyt

soxX

,

cyt

DRA0304_

1

aq_1811

aq_1810

aq_1809

aq_1807

aq_1806

aq_1803

aq_1802

aq_1800

aq_1799

aq_1798

aq_1797

aq_232

aq_234

aq_235

TTC1760

aq_979

TTC1045

COG3258,

soxD

Sulfite

oxidase

Sulfur oxidation protein

Sulfide DH

Sulfurtransferase

Cytochromes

Cytochrome

Thioredoxin

COG4911

**Fig. 2S. Operon responsible for oxidation of reduced sulfur compounds; Operon structure in *Thermus* similar to operon in *Aquifex***

**Influence of amino acid composition on phylogenetic reconstructions**

Sequence compositional bias is known to affect phylogeny reconstruction {Felsenstein, 2004 #162}. To test the level of this effect on our phylogenetic reconstructions, each alignment was bootstrapped 1000 times by alignment columns. 20-element amino acid frequency vectors (with gaps ignored) were computed for all sequences in each bootstrap sample. A matrix of Euclidean distances was computed for all vectors and 1000 neighbor joining trees (one per bootstrap sample) were built using NEIGHBOR program of PHYLIP package. Sequence bipartitions in these trees were analyzed using CONSENSE program of PHYLIP package. Probabilities (relative frequencies) for the following partitions were recorded:

*P*_0_ (TT,DR)

*P*_1_ (TT,DR,meso)

*P*_2_ (TT,DR,thermo)

*P*_3_ (TT,meso)

*P*_4_ (TT,thermo)

*P*_5_ (DR,meso)

*P*_6_ (DR,thermo)

where "meso" stays for any combination of mesophiles excluding thermophiles and "thermo" stays for any combination of thermophiles excluding mesophiles. Depending on the results of sequence-based phylogenetic reconstruction, support for "agreeing" and "disagreeing" composition-based bipartitions were computed as follows:

phylogeny *P*_agree_ *P*_disagree_

((TT,DR),meso) *P*_1_ *P*_2_

((TT,DR),thermo) *P*_2_ *P*_1_

(TT,thermo) *P*_4_ max(*P*_0_,*P*_1_,*P*_3_)

(DR,thermo) *P*_6_ max(*P*_0_,*P*_1_,*P*_5_)

If neither of the support probabilities exceeded 1/3, the composition-based tree was considered unresolved in regards to the clades in question; otherwise, a log-odds ratio for the agreement between sequence-based phylogeny and composition-based similarity was computed as

*L* = log_10_(*P*_agree_/*P*_disagree_)

If the value of *L* exceeded 0.5 (i.e. *P*_agree_/*P*_disagree_ > 3.16), we recorded it as a case of strong agreement between compositional bias and sequence-based phylogenetic reconstruction; the lack of such agreement was registered otherwise.

| Sequence-based phylogeny | No. of trees | agreement with AA bias |
| --- | --- | --- |
| ((TT,DR),thermo) | 29 | 5 |
| (TT,thermo) | 26 | 5 |
| (DR,thermo) | 1 | 0 |

Altogether, even at the very low support threshold of 33%, only 10 of the 56 families that group TT and/or DR with thermophiles showed preference for the same clustering by composition. Thus, we conclude that more than 80% of our reconstructions are likely not to be influenced by common compositional bias in TT and other thermophilic prokaryotes.

**Table 2S.** Genes coding for replication, repair and recombination functions^A^ in *E. coli,* *D. radiodurans* and *T. thermophilus*

| **Pathway^A^** | **Protein description and comments^B^** | **EC** | **DR** | **TT** | **COG and Commet (BLAST result)** |
| --- | --- | --- | --- | --- | --- |
| mMM? | Adenine-specific DNA  methylase | YhdJ | DRC0020 | TTC0041, TTC1880 | COG0863, some gamma do not have this gene |
| DR | O-6-methylguanine DNA methyltransferase | Ogt, YbaZ | -  DR0428 | TT1199  - | COG0350,  COG3695 |
| DR | 8-oxo-dGTPase. D.r. encodes additional 17 paralogs; only some predicted to function in repair | MutT | DR0261 | 5 paralogs: TTC0160, TTC0511, TTC1519, TTC1584, TTC1859 | COG0494 |
| DR, BER | 3-methyladenine DNA glycosylase II; DR2584 is of eukaryotic type | AlkA | DR2584,  DR2074 | TTC1654 | COG0122,  COG2094 |
| BER, MMY | 8-oxoguanine DNA glycosylase & AP-lyase, A-G mismatch DNA glycosylase | MutY | DR2285 | TTC1535 | COG1194 |
| BER | Endonuclease III & thymine glycol DNA glycosylase; DR0928 and DR2438 are of archaeal type and DR0289 is close to yeast protein | Nth | DR2438, DR0289, DR0928 | TTC1892 | COG0177 |
| BER | Formamidopyrimidine & 8-oxoguanine DNA glycosylase | MutM/Fpg | DR0493 | TTC1454 | COG0266 |
| BER | Endonuclease V | Nfi (YjaF) | DR2162 | TTC0982 | COG1515, some gamma do not have this gene |
| BER | DNA polymerase I | PolA_1_2 | DR1707 | TTC0690 | COG0258+COG0749 |
| BER | Uracil DNA glycosylase; DR0689 is a likely horizontal transfer from a eukaryote or a eukaryotic virus | Ung | DR0689  DR1663 | -  - | COG0692,  No COG |
| BER | G/T mismatch-specific thymine DNA glycosylase, distantly related to DR1751; Present as a domain of many multidomain proteins in many eukaryotes | Mug (ygjF) | DR0715 | - | COG3663 many gamma do not have this gene |
| BER | Uracil DNA glycosylase | - | DR1751 | TTC0366, TTC0784 (archaeal) | COG1573 |
| BER | Exodeoxyribonuclease III | XthA | DR0354 | - | COG0708 |
| NER, BER | Predicted ATP-dependent protease | Sms (RadA) | DR1105 | TTC0173 | COG1066 |
| NER | Transcription repair coupling factor; helicase | Mfd | DR1532 | TTC0533 | COG1197 |
| NER | ATPase, DNA binding | UvrA | DR1771, DRA0188 | TTC1075 | COG0178 |
| NER | Helicase | UvrB | DR2275 | TTC1531 | COG0556 |
| NER | Nuclease | UvrC | DR1354 | TTC1182 | COG0322 |
| NER, mMM, SOS | helicase II; initiates unwinding from a nick; DR1572 has a frameshift | UvrD,  BS_yvgS | DR1775,  DR1572 | TTP0191, TTC0638, TTC1062;  - | COG0210,  COG3973 |
| mMM, VSP | predicted ATPase | MutL | DR1696 | TTC0959 | COG0323 |
| mMM, VSP | ATPase; DR1039 has a frameshift | MutS | DR1976,  DR1039 | TTC1282 | COG1193,  COG0249 |
| MM | Exonuclease VII, large subunit | XseA/ nec7 | DR0186 (~420aa) | - | COG1570 |
| MM | Exonuclease VII, small subunit | XseB | DR2586 (~80aa) | - | COG1722 |
| RER | Exonuclease subunit, Predicted ATPase | SbcC | DR1922 | TTC0922 | COG0419 |
| RER | Exonuclease | SbcD | DR1921 | TTC0923 | COG0420 |
| RER, SOS | Recombinase; ssDNA-dependent ATPase, activator of LexA autoproteolysis | RecA | DR2340 | TTC1466 | COG0468 |
| RER | Helicase/exonuclease; Contains three additional N-terminal helix-hairpin-helix DNA-binding modules; closely related to RecD from B.subtilis and Chlamydia | RecD | DR1902 | - | COG0507 |
| RER | Predicted ATPase; required for daughter-strand gap repair | RecF | DR1089 | TTC1721 | COG1195 |
| RER | Holliday junction-specific DNA helicase; branch migration inducer | RecG | DR1916 | TTC0902 | COG1200 |
| RER | Single-stranded DNA-specific exonuclease | RecJ | DR1126 | TTC0803 | COG0608 |
| RER | Predicted ATPase | RecN | DR1477 | TTC1161 | COG0497 |
| RER | Required for daughter-strand gap repair | RecO | DR0819 | TTC0258 | COG1381 |
| RER | Helicase; suppressor of illegitimate recombination | RecQ | DR1289,  DR2444 | TTP0128_3  (TTP0128_1 is DnaQ, TTP0128_2 is DinG  TTC0323??(C-terminal domain?) | COG0514,  No COG |
| RER | Required for daughter-strand gap repair | RecR | DR0198 | TTC1236 | COG0353 |
| RER | Holliday-junction-binding subunit of the RuvABC resolvasome | RuvA | DR1274 | TTC1696 | COG0632 |
| RER | Helicase subunit of the RuvABC resolvasome | RuvB | DR0596 | TTC0038 | COG2255 |
| RER | Endonuclease subunit of the RuvABC resolvasome | RuvC | DR0440 | TTC0725 | COG0817 |
| MP | Polymerase subunit of the DNA polymerase III holoenzyme | DnaE | DR0507 (no inteins) | TTC1806 (two inteins) | COG0587 |
| MP | 3’-5’ exonuclease subunit of the DNA polymerase III holoenzyme | DnaQ | DR0856 | TTP0128_1  (TTP0128_2 is DinG, TTP0128_3 is RecQ) | COG0847 |
| MP | DNA ligase | LigA  yicF | DR2069 | TTC0732 | COG0272 |
| MP | Single-strand binding protein; D. radiodurans R1 has three incomplete ORFs corresponding to different fragments of the SSB | Ssb | DR0099 | TTC1741 | COG0629 |
| SOS | Transcriptional regulator, repressor of the SOS regulon, autoprotease | LexA | DRA0344,  DRA0074 | -  - | COG1974,  No COG |
| VSP? | Uncharacterized proteins related to vsr | YcjD | DR0221,  DR2566 | -  - | COG2852,  No COG |
| ? | Uncharacterized family of presumably metal-dependent enzymes | Bs_DinB | 13 homologs (only 3 in COG) | - | COG2318 |
| DR | xantosine triphosphate pyrophosphatase, prevents 6-N-hydroxylaminopurin mutagenesis | HAM1/YggV | DR0179 | TTC1290 | COG0127 |
| NER | UV-endonuclease; Activity was characterized in Neurospora | Uve1/BS_YwjD | DR1819 | TTP0052 | COG4294 |
| NER | DNA or RNA helicase of superfamily II; also predicted nuclease; Contains an additional McrA nuclease domain | YejH/rad25 | DRA0131_1_2 | - | COG1061 |
| ? | Topoisomerase IB | - | DR0690 | - | COG3569 |
| ? | 3'->5' nuclease; Related to baculoviral DNA polymerase exonuclease domain | - | DR1721 | - | No COG |
| ? | Ro RNA binding protein; Ribonucleoproteins complexed with several small RNA molecules. Involved in UV-resistance in Deinococcus | - | DR1262 | - | No COG |
| ? | Predicted nuclease and Zinc finger domain containing protein. An ortholog is present in Pseudomonas aeruginosa | - | DR1757 | - | No COG |
| ? | Mrr-like nuclease;  Restriction endonuclease | -  Mrr | DR1877;  DR0508, DR0587 | -  - | COG1787,  COG1715 (most gamma do no have this gene) |
| New  Protein (Battista) | Single strand DNA-binding protein; protects 3’ ends from nuclease degradation | - | DR0423 (DdrA) | TTC1923 | COG4712 |
| New  Protein (Lena) |  |  | DR0070 (DdrB) | - | No COG |
| New  protein |  |  | DR0003(DdrC) | - | No COG |
| New  protein |  |  | DR0326 (DdrD) | - | No COG |

^A^Based largely on Makarova *et al*., Microbiol Mol Biol Rev. 2001 Mar;65(1):44-79, with modifications.

^B^Abbreviations of DNA repair pathways: DR- direct damage reversal; BER – base excision repair; NER – nucleotide excision repair; mMM – methylation-dependent mismatch repair; MMY – MutY - dependent mismatch repair; VSP – very short patch mismatch repair; RER – recombinational repair, SOS – SOS repair; MP – multiple pathways; putative, unconfirmed repair pathways are designated by a question mark.

^C^The gene names are from *E. coli*, whenever an *E. coli* ortholog exists, or from *B. subtilis* (with the prefix BS_).

**Table 3S*.*** *E. coli* and *T. .thermophilus*, repair genes^A^ with no orthologs in *D. radiodurans*

| **Pathway^A^** | **Protein description and comments^B^** | **EC** | **DR** | **TT** | **COG and Commet (BLAST result)** |
| --- | --- | --- | --- | --- | --- |
| BER | 3-methyladenine DNA glycosylase I | Tag | - | - | COG2818 |
| VSP | strand-specific, site specific, GT mismatch endonuclease; fixes deamination resulting from Dcm | Vsr | - | - | COG3727 (most gamma do not have it) |
| RER | Endonuclease /Holliday junction resolvase | RusA (YbcP) | - | - | COG4570 |
| RER | Helicase/exonuclease | RecB | - | - | COG1074 |
| RER | Helicase/exonuclease | RecC | - | - | COG1330 |
| DR | O6-methylguanine-DNA methyltransferase; transcription activator/repressor | Ada_1_2 | - | TTC1199 (second domain only, COG0350) | COG2162+COG0350 |
| DR, BER(?) | Unknown | AlkB | - | - | COG3145 |
| DR | dUTPase | Dut | - | - | COG0756 |
| DR | dCTP deaminase | Dcd | - | TTC1864 | COG0717 |
| BER | Endonuclease IV | Nfo | - | TTC0482 | COG0648, some gamma do not have it |
| DR | Photolyase | PhrB | - | TTP0058 | COG0415 |
| mMM | Endonuclease | MutH | - | - | COG3066 |
| mMM | GATC-specific N-6 adenine methlytransferase; imparts strand specificity to mismatch repair | Dam | - | - | COG0338 |
| SOS | DNA polymerase II | PolB | - | - | COG0417 |
| mMM, RER | Exodeoxyribonuclease I | SbcB | - | - | COG2925 |
| mMM | site-specific C-5 cytosine methlytransferase; VSP is targeted toward hotspots created by dcm | Dcm | - | - | COG0270, some gamma do not have it |
| MM, RER | Specific function unknown (predicted nucleotidyltranferase) | DinP | - | - | COG0389 |
| RER | exonuclease VIII | RecE | - | - | No COG, most gamma do not have it |
| RER | annealing protein | RecT | - | - | COG3723 most gamma do not have it |
| SOS | predicted helicase; SOS inducer | DinG | - | TTP0128_2 (TTP0128_1 is DnaQ, TTP0128_3 is RecQ) | COG1199 |
| SOS | Error-prone DNA polymerase; in conjunction with umuD and recA, catalyzes translesion DNA synthesis | UmuC | - | - | COG0389 |
| SOS | In conjunction with umuC and recA, facilitates translesion DNA synthesis; autoprotease | UmuD | - | - | COG1974 |
| BER | Predicted acyltransferase; predicted DNA-binding protein | RadC | - | - | COG2003 |
|  | Uncharacterized protein predicted to be involved in DNA repair | [ygbT](http://www.ncbi.nlm.nih.gov/COG/new/release/bluz.cgi?cog=ygbT&ygbT) | - | TTP0196, TTC1170 | COG1518 (archaeal origin?) |
|  | Uncharacterized protein predicted to be involved in DNA repair | [BH0342](http://www.ncbi.nlm.nih.gov/COG/new/release/bluz.cgi?cog=BH0342&BH0342) | - | TTP0101, TTP0195 | COG1343 |
|  | RecB family exonuclease | [BH0340](http://www.ncbi.nlm.nih.gov/COG/new/release/bluz.cgi?cog=BH0340&BH0340) | - | TTP0136, TTP0197 (both archaeal origin?) | COG1468 |

Footnotes for Supplemental Table II are as for Supplemental Table I.

**Table 4S.** Stress response-related genes in *D. radiodurans and T.thermophilus*

| **Type of stress** | **Protein description and comments** | **Gene name** | **DR gene_ID** | **TT gene_ID** | **COG and comment** |
| --- | --- | --- | --- | --- | --- |
| Heat/ general | Heat Shock Protein 10, molecular chaperone | GroL | DR0607 | TTC1714 | COG0459 |
| Heat/ general | Heat Shock Protein 20, molecular chaperone | GrpE | DR0128 | TTC1126 | COG0576 |
| Heat/ general | Heat Shock Protein 60, molecular chaperone | GroS | DR0606 | TTC1713 | COG0234 |
| Heat/ general | Heat Shock Protein 70, molecular chaperone | DnaK | DR0129 | TTC1127 | COG0443 |
| Heat/ general | Hsp70 chaperone co-factor | DnaJ | DR0126; DR1424 | TT?;  TTC1812 | COG2214;  COG0484 |
| Heat/ general | Small heat-shock protein | IbpA/ IbpB | DR1114 DR1691 | TTC1116 TTC1120 TTC1257 TTC0203 | COG0071 |
| Heat | Related to heat shock protein, HSLJ; DR1940 contains 3 repeats of this domain | HSLJ | DR2056 DR1940 | - | COG3187 |
| General | ATPase subunit of Clp protease | ClpA/ ClpB | DR0588 DR1046 DR1117 | TTC1123 | COG0542 |
| General | ATPase subunit of Clp protease | ClpX | DR1973; DR0202 (+4 paralogs) | TTC0251 | COG1219;  COG0563 |
| General | ATP-dependent protease with chaperone activity | ClpP | DR1972 | TTC0250 | COG0740 |
| General | ATP-dependent Lon serine protease | Lon | DR1974 DR0349;  DR2189 | TTC0418 TTC0746; | COG0466;  COG2802 |
| General | ATP-dependent serine protease | Sms | DR1105 | TTC0173 | COG1066 |
| General | Do serine protease, with regulatory PDZ domain | HtrA | DR0327 DR0745 DR1599 DR1756 DR0984 DR0300 | TTC0417 TTC0956  TTC1905 | COG0265 |
| General | Tail-specific periplasmic serine protease | Prc | DR1308 DR1491 DR1551 | TTC0929 | COG0793 |
| General | Membrane-associated Zn-dependent protease I | YaeL | DR1507 | TTC0503 TTC1159 (short, check!) | COG0750 |
| General | ATP-dependent Zn protease | FtsH | DR0583 DR1020 DRA0290 | TTC0035 TTC1128 | COG0465 |
| General | Predicted Zn-dependent proteases (possible chaperones) | HtpX | DR01904;  DR0194 | -;  TTC0540 | COG0501;  COG2738 |
| General | Membrane chaperone | SugE | DR1004 DR1005 | TTC0565 | COG2076 |
| General | Diadenosine tetraphosphate (Ap4A) hydrolase, HIT family, cell cycle regulation | Hit | DR1621 | TTC1253 | COG0537 |
| General | Zn-binding (lipo)protein of the ABC type Zn transport system (surface adhesin A) | YebL | DR2523 | TTC0227 | COG0803 |
| General | GTPase, protease modulator | HflX | DR0139 DR0646 | TTC0198 | COG2262 |
| ? | Fibronectin-binding protein, function unknown | BS_yloA | DR0559 | TTC0243 | COG1293 |
| General | General stress protein, related to thioredoxin | BS_ytxJ | DR1832 |  | No COG |
| General | Protease I, related to general stress protein 18, ThiJ superfamily protein | ThiJ | DR1199 DR0491 | TTC1088 TTC1585 | COG0693 |
| General | Universal stress protein, nucleotide-binding | UspA | DR2363 DR2132 | TTC0539 TTC1692 TTC1794 TTC1633 | COG0589 |
| Starvation | Guanosine polyphoshate (ppGpp) pyrophosphohydrolase/synthetase; No RelA counterpart like in Gram+ bacteria | SpoT | DR1838 | TTC1355 | COG0317 |
| ? | Histone-like DNA-binding protein | HupA | DRA0065 | TTC0984 | COG0776 |
| ? | Haemoglobin-like flavoprotein | Hmp | DRA0243 |  | No COG |
| Starvation | ppGpp regulated growth inhibitor | MazF | DR0417 DR0662 | - | COG2337 |
| Starvation | Regulatory protein, *MazF* antagonist | MazE | DR0416 | TTP0184?? Wrong hit? | COG2336 |
| ? | Phosphatase of ppGpp | Ppx | DRA0185 | TTC0636 | COG0248 |
| Starvation | Starvation inducible DNA-binding protein | Dps | DR2263 DRB0092 | - | COG0783 |
| Osmotic | Large conductance mechanosensitive channel | Mscl | DR2422 | TTC0261 | COG1970 |
| Osmotic | Membrane protein | Yggb | DR1995 DR0211 | TTC1353 | COG0668 |
| Osmotic | Osmosensitive K+ channel Histidine kinase sensor domain | KdpD | DRB0088 | - | COG2205 |
| Osmotic | Potassium uptake system, NAD-binding component | TrkA | DR1666 | TTC0810 (long), symmetrical DR2336 (COG1226) | COG0569 |
| Osmotic | Potassium uptake system component | TrkH/ TrkG | DR1667 DR1668 | TTC0809 no DR symmetrical hits | COG0168 |
| Osmotic | Proline/ glycine betaine ABC-type transport, permease subunit | ProW | DRA0138 DRA0139 | - | COG1174 |
| Osmotic | Proline/ glycine betaine ABC-type transport, ATPase subunit | ProV | DRA0137 | - | COG1125 |
| Osmotic | Proline/ glycine betaine ABC-type transport, periplasmic binding subunit | YehZ | DRA0135 | - | COG1732 |
| Phage | Phage shock protein A, controls membrane integrity | PspA | DR1473 | TTC1616 | COG1842 |
| Alkaline | Alkaline shock protein, function unknown | BS_yloU/ BS_yqhY | DR2068 DR0389 | TTC0757  TTC1771 | COG1302 |
| Cold | Cold shock protein, OB fold nucleic acid binding protein | Csp | DR0907 | TTC1626 TTC1811 | COG1278 |
| ? | Competence damage protein, mitomycin-induced, function unknown | CinA | DR2838_1_2 | TTC1468_1_2 | COG1058/COG1546 |
| Oxidative | Catalase; DRA0259 has C-terminal proteinase I-like domain | KatE | DR1998 DRA0259 | - | COG0753 |
| Oxidative | Catalase; Eukaryotic type, presumably acquired from nitrogen–fixing bacteria | KatA S.pombe | DRA0146 | - | COG0753 |
| Oxidative | Peroxidase; Yet present only in plant Polyporaceae sp. | NA | DRA0145 | - | COG2837 |
| Oxidative | Superoxide dismutase Mn or Fe dependent | SodA | DR1279 | TTC0189 | COG0605 |
| Oxidative | Superoxide dismutase Cu/Zn dependent | SodC | DR1546 DRA0202 | - | COG2032 |
| ? | Ferric uptake regulation protein | Fur | DR0865 | TTC0925  TTC1639  TTC1730 | COG0735 |
| Oxidative | Antioxidant type thioredoxin fold protein | Bcp | DR0846 DR1208  DR1209 | TTC0933  TTP0168 | COG1225 |
| Oxidative | Protein involved in alkylperoxide and oxidative stress response, osmotically induced protein | OsmC | DR1538 DR1857 | TTC1261 | COG1764 |
| Oxidative | Protein involved in alkylperoxide and oxidative stress response, osmotically induced protein | YhfA | DR1177 | TTC1007 | COG1765 |
| Oxidative | Peptide methionine sulfoxide reductase PMSR | MsrA | DR1849 | TTP0095 | COG0225 |
| Oxidative/ detoxication | Thiol-alkyl hydroperoxide reductases | AhpC | DR2242 DR1209 | - | COG0450 |
| Oxidative/ detoxication | Thioredoxin reductase/alkyl hydroperoxide reductase | Ahpf/ TrxB | DR1982 DR2623 DR0412 DRB0033_2 | TTC0003 TTC0096 TTC0853 TTC1555;  - | COG0492;  COG2072 |
| Oxidative/ detoxication | Glutaredoxin | GrxA | DR2085 DRA0072 | - | COG0695 |
| Detoxication | Cytochrome P450 (uses O_2_) | BS_cypA or TerA of Alcalige-nes | DR2473 DR2538 DR1723 DRA0186 DRC0041 DRC0001 | TTP0059 | COG2124 |
| Detoxication | Function unknown, involved in tellurium resistance response in *Alcaligenes* | TerB of Alcalige-nes | DR2220 | - | COG3793 |
| Detoxication | Function unknown, membrane protein | TerC of Alcalige-nes | DR2226; DR1187 DRB0131 | -  TTC0142 TTC0750 | COG2899;  COG0861 |
| Detoxication | Toxic anion resistance protein , possibly tellurite resistance | BS_yceH | DR1127 | - | COG3853 |
| Toxins/ general | Chemical damaging agent resistance; In *B.s.* it is involved in low temperature and salt stress response | BS_Scp2 | DR2225 DR2221 DR2224 DR2223_1 DRA0057_1 DR2217_1_2 | - | COG2310 |
| Toxins | Arsenate oxidoreductase (arsC-like Rodanese protein) | ArsC | DRA0123; DR0136 | TTC1502 TTC0816 | COG0394;  COG1393 |
| Desiccation | Desiccation protectant, LEA14 family | NA | DR1372 | - | No COG |
| Desiccation | Desiccation-related protein from *Craterostigma plantagineum*; Found to date only in plants | NA | DRB0118 | TTP0170 | No COG |
| Desiccation | LEA76 family desiccation resistance protein | NA | DR0105 DR1172 | -  - | No COG |
| Drugs | Erythromycin esterase | BS_ybfO | DRA0345 DR2257 | - | COG2312 |
| Drugs | BacA bacitracin resistance protein, undecaprenol kinase | BacA | DR0454 | TTC1814 | COG1968 |
| Drugs | Streptomycin resistance protein, streptomycin phosphotransferase | StrA of Strepto-myces | DR0455 | - | COG3570 |
| Drugs | Antibiotic (aminoglycoside); Kinase family protein | BS_ycbJ | DR0066; DRA0194; DR0394; DR0669 | -; -; -; - | COG3231; COG3173; COG2334;  no COG |
| Drugs | 5-Nitroimidazole antibiotic resistance protein; distantly related to pyridoxamine phosphate oxidase, PDXH | NimABCD of Bacteroides | DR0842 | - | COG3467 |
| Drugs | Function unknown, involved in multidrug resistance | BS_bmrU | DR2234 DR1363 DR1560 | TTC1957 | COG1597 |
| Drugs | Thiophen and furan oxidation, predicted GTPase | ThdR | DR1016 | TTC0562 | COG0486 |
| Drugs | tunicamycin resistance protein, predicted ATPase | BS_tmrB | DR1419 | - | No COG |
| Drugs | Beta-lactamase | BS_penP | DRA0241 DR0433 (+DR1985) | TTC0404 | COG2367 |
| Drugs | Function unknown, lactam utilization protein | YbgL | DRA0284 | TTP0137 | COG1540 |
| Drugs | Lactoylgluthation lyase, phosphomicin resistance protein | GloA | DR1695 DR2022 DR2104 DR2208 DR0109 DRA0224 (+DR1341 and DR0670) | TTC0024 TTC0596_2 TTC1190 TTC1694 | COG0346 |
| Drugs | Induced by Vancomycin in *Enterococcus faecalis* | BS_yoaR | DR1619 DR0009 DR0025 | TTC1933 | COG2720 |
| Drugs | aminoglycosid N3-acetyltransferase; Present in many other bacteria | BS_yokD | DR2034 DR0599 | - | COG2746 |
| Drugs | Function unknown, homologs of microcine C7 resistance protein MccF | BS_yocD | DR2000 | - | COG1619 |
| Drugs | Function unknown, Penicillin tolerance protein | lytB | DR2164 | TTC1983 | COG0761 |
| Drugs | 2-nitropropane dioxygenase | BS_yrpB | DR2545 | TTC1901 | COG2070 |
| Drugs | phosphinothricin aminoacetyltransferase | BS_ywnH | DR1182 | - | COG1247 |
| **Genes that are absent in *D. radiodurans*** | | | | | |
| Starvation | Carbon starvation-induced protein, membrane | CstA | - | - | COG1966 |
| General | ATPase subunit of clp photolytic system | ClpY (HslU) | - | TTC0264 | COG1220 |
| Oxidative | Glutathione peroxidase | BtuE/ BS_bsaA | - | - | COG0386 |
| Oxidative | Catalase (peroxidase I) | KatG | - | - | COG0376 |
| Oxidative | Mn-containing catalase |  | - | TTC1872 | COG3546 |
| Heat/ general | Heat Shock Protein 90, molecular chaperone | HtpG | - | - | COG0326 |
| Heat/ general | *DnaK* suppressor protein | DksA | - | - | COG1734 |
| Heat/ general | Transcriptional regulator of heat shock genes | HrcA | - | TTC1948 | COG1420 |
| Acid | unknown | YajQ | - | - | COG1666 |
| Osmotic | Trehalose-6-phosphate synthase | OtsA | - | - | COG0380 |
| Osmotic | Trehalose-6-phosphatase | OtsB | - | - | COG1877 |

Note: Abbreviations in the phylogenetic patterns and gene descriptions are as in Tab

**Fig. 3S. A genome dot-plot comparison for homologous megaplasmids of two *T.thermophilus* strains *(HB8 and HB27)***

Each dot represents a pair of probable orthologs defined as reciprocal BLAST best hits with E-value <0.001.

**Reconstruction of the gene set of the Last Bacterial Common Ancestor**

To infer the gene set for the Last Bacterial Common Ancestor (LBCA), we implemented an empirical parsimony procedure based on the table below. Presence or absence of COG members in a list of organisms was used, in an hierarchical manner, to determine whether this COG was present in the last common ancestor of the group. For example, a COG was assigned to the common ancestor of α-Proteobacteria if it is represented in at least four of 7 species of this taxon (Atu, Sme, Bme, Mlo, Ccr, Rco and Rpr). To qualify for the common ancestor of all proteobacteria, a COG should be in the root of at least two of three proteobacteria groups (α-Proteobacteria, βγ-Proteobacteria and ε-Proteobacteria); to be considered an LBCA gene, a COG must be assigned to the ancestral nodes of at least two of the four major bacterial clades (Cyanobacteria+ Actinobacteria+ Deinococcus, Fusobacterium+ Firmicutes, Proteobacteria and Chlamydia+ Spirochaetes). Several redundant genomes (e.g., additional strains of *E. coli*) and species thought to be extremely prone to horizontal gene transfer (*A. aeolicus* and *T. maritima*) were excluded.

**Hierarchy of bacterial lineages used for inferring the LBCA gene set**

| Bacteria  ≥2 (of 4) | Cyanobacteria+ Actinobacteria+ Thermus+ Deinococcus  ≥2 (of 3) | Cyanobacteria  ≥1 (of 2) | Syn |
| --- | --- | --- | --- |
|  |  |  | Nos |
|  |  | Actinobacteria  ≥1 (of 2) | Cgl |
|  |  |  | Mtu |
|  |  | Thermus+Deinococcus  ≥1 (of 2) | Tth |
|  |  |  | Dra |
|  | Fusobacterium+ Firmicutes  ≥2 (of 4) |  | Fnu |
|  |  |  | Cac |
|  |  | Bacilli  ≥4 (of 6) | Lla |
|  |  |  | Spn |
|  |  |  | Sau |
|  |  |  | Lin |
|  |  |  | Bsu |
|  |  |  | Bha |
|  |  | Mollicutes  ≥1 (of 2) | Uur |
|  |  |  | Mpn |
|  | Proteobacteria  ≥2 (of 3) | βγ-Proteobacteria  ≥6 (of 10) | Eco |
|  |  |  | Ype |
|  |  |  | Sty |
|  |  |  | Buc |
|  |  |  | Vch |
|  |  |  | Pae |
|  |  |  | Hin |
|  |  |  | Pmu |
|  |  |  | Xfa |
|  |  |  | Nme |
|  |  |  | Rso |
|  |  | ε-Proteobacteria  ≥1 (of 2) | Hpy |
|  |  |  | Cje |
|  |  | α-Proteobacteria  ≥4 (of 7) | Atu |
|  |  |  | Sme |
|  |  |  | Bme |
|  |  |  | Mlo |
|  |  |  | Ccr |
|  |  |  | Rpr |
|  |  |  | Rco |
|  | Chlamydia+ Spirochaetes  ≥2 (of 2) | Chlamydia  ≥1 (of 2) | Ctr |
|  |  |  | Cpn |
|  |  | Spirochaetes  ≥1 (of 2) | Tpa |
|  |  |  | Bbu |

Abbreviated species names are from the COG database (http://www.ncbi.nlm.nih.gov/COG/).

**Gene identifiers and species names for figure 6**

| Gene_ID | Species |
| --- | --- |
| MJ1478 | Methanocaldococcus jannaschii DSM 2661 |
| PH0300 | Pyrococcus horikoshii OT3 |
| aq_1333 | Aquifex aeolicus VF5 |
| TM0197 | Thermotoga maritima MSB8 |
| TTC0106 | Thermus thermophilus HB27 |
| AF1321 | Archaeoglobus fulgidus DSM 4304 |
| Cj0118 | Campylobacter jejuni subsp. jejuni NCTC 11168 |
| HP1182 | Helicobacter pylori 26695 |
| DR0480 | Deinococcus radiodurans R1 |
| PA1192 | Pseudomonas aeruginosa PAO1 |
| HI1371 | Haemophilus influenzae Rd KW20 |
| PM0606 | Pasteurella multocida subsp. multocida str. Pm70 |
| ydaO | Escherichia coli K12 |
| VC1432 | Vibrio cholerae O1 biovar eltor str. N16961 |
| NMB1023 | Neisseria meningitidis MC58 |
| XF0569 | Xylella fastidiosa 9a5c |
| mlr0047 | Mesorhizobium loti MAFF303099 |
| MK1103 | Methanopyrus kandleri |
| VNG0190C | Halobacterium sp. NRC-1 |
| MTH1186 | Methanothermobacter thermautotrophicus str. Delta H |
| SSO0586 | Sulfolobus solfataricus P2 |
| APE0537 | Aeropyrum pernix K1 |
| MJ1157 | Methanocaldococcus jannaschii DSM 2661 |
| MA1974 | Methanosarcina acetivorans C2A |
| Ta1119 | Thermoplasma acidophilum DSM 1728 |
| PH1680 | Pyrococcus horikoshii OT3 |
| AF1595 | Archaeoglobus fulgidus DSM 4304 |
| APE2086 | Aeropyrum pernix K1 |
| all0982 | Nostoc sp. |
| slr0118 | Synechocystis sp. |
| DRA0175 | Deinococcus radiodurans R1 |
| RSc0113 | Ralstonia solanacearum |
| NMB2040 | Neisseria meningitidis MC58 |
| PA4973 | Pseudomonas aeruginosa |
| VC0061 | Vibrio cholerae O1 biovar eltor str. |
| thiC | Escherichia coli K12 |
| mll5795 | Mesorhizobium loti MAFF303099 |
| AGc4656 | Agrobacterium tumefaciens str. C58 |
| CC2029 | Caulobacter crescentus CB15 |
| BH1933 | Bacillus halodurans |
| BS_thiA | Bacillus subtilis |
| XF1888 | Xylella fastidiosa 9a5c |
| Cgl1275 | Corynebacterium glutamicum |
| Rv0423c | Mycobacterium tuberculosis |
| VNG0715G | Halobacterium sp. |
| TM0788 | Thermotoga maritima MSB8 |
| MTH1576 | Methanothermobacter thermautotrophicus str. Delta |
| MTH1543 | Methanothermobacter thermautotrophicus str. Delta |
| MJ1026 | Methanocaldococcus jannaschii DSM |
| MK0106 | Methanopyrus kandleri |
| AF2412 | Archaeoglobus fulgidus |
| PAB1930 | Pyrococcus abyssi GE5 |
| MA0261 | Methanosarcina acetivorans |
| SSO1324 | Sulfolobus solfataricus |
| PAE0333 | Pyrobaculum aerophilum str. IM2 |
| CAC3014 | Clostridium acetobutylicum |
| TTC0319 | Thermus thermophilus HB27 |
| aq_1204 | Aquifex aeolicus |
| PH1756 | Pyrococcus horikoshii OT3 |
| AF1904 | Archaeoglobus fulgidus |
| MA1093 | Methanosarcina acetivorans C2A |
| VNG1716G | Halobacterium sp. |
| MJ0476 | Methanocaldococcus jannaschii DSM 2661 |
| APE0345 | Aeropyrum pernix K1 |
| SSO0697 | Sulfolobus solfataricus |
| PAE1183 | Pyrobaculum aerophilum str. IM2 |
| Rv0722 | Mycobacterium tuberculosis |
| Cgl0528 | Corynebacterium glutamicum |
| TTC1310 | Thermus thermophilus HB27 |
| aq_1644 | Aquifex aeolicus |
| TM1482 | Thermotoga maritima MSB8 |
| DR2114 | Deinococcus radiodurans R1 |
| SA2030 | Staphylococcus aureus |
| lin2763 | Listeria innocua |
| BS_rpmD | Bacillus subtilis |
| L0424 | Lactococcus lactis |
| CAC3115 | Clostridium acetobutylicum |
| PM1397 | Pasteurella multocida |
| VC2578 | Vibrio cholerae O1 biovar eltor str. N16961 |
| rpmD | Escherichia coli K12 |
| PA4245 | Pseudomonas aeruginosa |
| NMB0160 | Neisseria meningitidis MC58 |
| RSc3001 | Ralstonia solanacearum |
| XF1170 | Xylella fastidiosa 9a5c |
| CC1266 | Caulobacter crescentus CB15 |
| msr0316 | Mesorhizobium loti MAFF303099 |
| RP641 | Rickettsia prowazekii str. Madrid E |
| Ta1250m | Thermoplasma acidophilum |
| VC2577 | Vibrio cholerae O1 biovar eltor str. N16961 |
| CC1267 | Caulobacter crescentus CB15 |
| AGc3525 | Agrobacterium tumefaciens str. C58 |
| mlr0318 | Mesorhizobium loti MAFF303099 |
| Cgl0529 | Corynebacterium glutamicum |
| Rv0723 | Mycobacterium tuberculosis |
| ML1840 | Mycobacterium leprae |
| TTC1309 | Thermus thermophilus HB27 |
| GSU2838 | Geobacter sulfurreducens |
| aq_1642 | Aquifex aeolicus |
| HP1301 | Helicobacter pylori 26695 |
| STH3056 | Symbiobacterium thermophilum |
| CAC3114 | Clostridium acetobutylicum |
| GK0125 | Geobacillus kaustophilus |
| BS_rplO | Bacillus subtilis |
| ABC0169 | Bacillus clausii |
| TM1481 | Thermotoga maritima MSB8 |
| TTE2273 | Thermoanaerobacter tengcongensis |
| Ta1249 | Thermoplasma acidophilum DSM 1728 |
| RP640 | Rickettsia prowazekii str. Madrid E |
| PH1755 | Pyrococcus horikoshii OT3 |
| MTH25 | Methanothermobacter thermautotrophicus str. |
| AF1903 | Archaeoglobus fulgidus |
| MA1094 | Methanosarcina acetivorans C2A |
| SSO0696 | Sulfolobus solfataricus |
| APE0343 | Aeropyrum pernix K1 |
| DR2115 | Deinococcus radiodurans R1 |
| sll1813 | Synechocystis sp. |
| all4198 | Nostoc sp. |
| NMB0161 | Neisseria meningitidis MC58 |
| RSc3000 | Ralstonia solanacearum |
| PA4244 | Pseudomonas aeruginosa |
| rplO | Escherichia coli K12 |
| SA2029 | Staphylococcus aureus |
| SPy0072 | Streptococcus pyogenes |
